# Accumulation and maintenance of information in evolution

**DOI:** 10.1073/pnas.2123152119

**Published:** 2022-08-29

**Authors:** Michal Hledík, Nick Barton, Gašper Tkačik

**Affiliations:** ^a^Institute of Science and Technology Austria, AT-3400 Klosterneuburg, Austria

**Keywords:** evolution, population genetics, information

## Abstract

Selection accumulates information in the genome—it guides stochastically evolving populations toward states (genotype frequencies) that would be unlikely under neutrality. This can be quantified as the Kullback–Leibler (KL) divergence between the actual distribution of genotype frequencies and the corresponding neutral distribution. First, we show that this population-level information sets an upper bound on the information at the level of genotype and phenotype, limiting how precisely they can be specified by selection. Next, we study how the accumulation and maintenance of information is limited by the cost of selection, measured as the genetic load or the relative fitness variance, both of which we connect to the control-theoretic KL cost of control. The information accumulation rate is upper bounded by the population size times the cost of selection. This bound is very general, and applies across models (Wright–Fisher, Moran, diffusion) and to arbitrary forms of selection, mutation, and recombination. Finally, the cost of maintaining information depends on how it is encoded: Specifying a single allele out of two is expensive, but one bit encoded among many weakly specified loci (as in a polygenic trait) is cheap.

## Significance

Through variation in fitness, selection accumulates and maintains information in the genomes of organisms. This process takes place over many generations, in populations that evolve stochastically due to finite size and random mutation. The information, which we quantify in bits, corresponds to the degree to which selection shapes the population composition, the DNA sequence, and the phenotype. We prove a general bound on the rate at which information can accumulate per generation. We find that both accumulation and maintenance of information are most efficient (require the least fitness variation per bit) when individual loci experience weak selection. This is relevant for selection on traits influenced by many small-effect loci—a common genetic architecture according to genome-wide association studies.

Throughout evolution, selection accumulates information in the genome. It guides evolving populations toward fitter phenotypes, genotypes, and genotype frequencies, which would be highly unlikely to arise by chance. This information—the degree to which selection can control the stochastic process of evolution—has been a long-standing subject of research (1–7), and relates to basic questions in evolutionary biology and genetics.

## 1. Introduction

### 1.1. How Well Can Selection Specify the Genotype and the Phenotype?.

The degree to which within- and between-species genetic variations are shaped by selection has been the subject of the neutralist–selectionist debate (8–11). Today, we know that much of the human genome is involved in various biochemical processes (12, 13), but this does not mean that it is strongly shaped by selection (14–16). Here we ask a related question in information-theoretic terms: How much information can selection accumulate and maintain in the genome? Much of the sequence is to some degree random, and, given its size, $l\approx 3\times 10^{9}$ base pairs, it likely contains far less information than the maximum conceivable $6\times 10^{9}$ bits of information. A similar question has been raised in the context of origin of life: Given high mutation rates, how much information could be maintained in the genome of early organisms (2)?

Analogous questions can be asked about the phenotype. How many traits can selection optimize? It is easy to list a large number of potentially relevant traits: Take the expression of all genes in all cell types and conditions, or regulatory interactions between pairs of genes. For a fit organism, these traits need to be specified with some precision, and this precision is likely limited (even if it is, to some degree, facilitated by correlations among traits). For example, a study of selective constraint on human gene expression (17) gave evidence of constraint, but, overall, this seems weak. Given the large number of possibly important phenotypes, how precisely can selection specify them?

### 1.2. Quantifying Genetic Information.

An established method in bioinformatics quantifies the information content of a short genomic motif, such as a binding site, by comparing an alignment of its instances across the genome to the genomic background (18, 19). Our definition of genetic information is mathematically similar, but aims to apply more generally (to large regions without multiple instances available). It is therefore based in theoretical population genetics rather than sequence data analysis. A key related concept is the repeatability of evolution (20, 21). Evolution is stochastic due to genetic drift and mutation, but selection can reduce the space of possible outcomes. For example, suppose that, in a sequence of length *l*, *n* sites are under strong selection for specific nucleotides. By fixing those nucleotides, selection will accumulate 2*n* bits of information. Meanwhile, the remaining *l* – *n* sites will be occupied by random nucleotides, and, if a replicate population evolves under identical conditions, the *l* – *n* nucleotides will likely be different. Therefore, our concept of information in a sequence is inversely related to how differently it could have evolved under identical conditions.

In general, however, the information content of the genome cannot be quantified by simply counting the sites that are under selection. A single bit of information can be spread across many loci under weak selection—a phenomenon particularly relevant when selection acts on polygenic traits, long recognized in quantitative genetics and described by the infinitesimal model (22, 23). Polygenicity and weak selection also resolve the apparent contradiction between the variety of phenotypes, or biochemical processes involving the DNA, and the lack of strong selective constraint on all of them. Selection might act on a small number of high-level traits, which are influenced by large numbers of loci spread across the genome [described by the omnigenic model (24)], which experience only weak selection individually.

In Section 2, we define information on three levels—the population state (genotype frequencies), the genotype, and the phenotype. There are simple inequalities between the three levels. This means that the upper bound on information accumulation rate, which we prove at the population level, also implies a bound at the genotype and phenotype levels. We use the Kullback–Leibler [KL] divergence, a central quantity in information theory (25), to quantify the difference between their actual distribution and their corresponding neutral distribution.

Notably, the neutral phenotype distribution corresponds approximately to the phenotype distribution among random DNA sequences. Recent work with random mutant libraries suggests that, for some phenotypes, this distribution is accessible experimentally [gene expression driven by random promoters (26–28) or enhancers (29)]. Any departure from this neutral distribution amounts to accumulation of information.

### 1.3. Cost of Information.

After defining what genetic information means, we ask how quickly it can accumulate and how much of it can be maintained. We look for answers in terms of the cost of selection—the amount of relative fitness variation in a population. This cost, traditionally measured as the relative fitness variance or the genetic load, is itself limited. In a population with constant size, relative fitness is proportional to the expected number of offspring, and the number of offspring can only vary between zero and the reproductive capacity of the organism.

We rely on an information-theoretic measure of cost of selection, which is itself upper bounded by the relative fitness variance and genetic load but has favorable mathematical properties. It relates the cost of selection to the KL cost of control (30–32), or the thermodynamic power (33).

The relationship between information accumulation rate and the cost of selection has been studied by Kimura (1) and, later, Worden (3), MacKay (4), and Barton (7). In Section 3, we discuss these works in more detail and derive a more general bound. The problem of maintenance has been studied by Eigen (2), Watkins (5), and Peck and Waxman (6). We discuss these in Section 4 and present example calculations that suggest general trends in the amount of information that can be maintained per unit cost.

## 2. Quantifying Genetic Information

The measures of information studied in this paper are based on comparisons between the distributions of various variables under selection versus neutrality. The focus on probability distributions accounts for the stochasticity of evolution, and the difference between the distributions with and without selection corresponds to the control that selection exerts on evolution. We quantify this difference in bits, using the KL divergence (25)

[1] $$D(U)=\sum_{u}\psi^{U}(u)\log_{2}\frac{\psi^{U}(u)}{\varphi^{U}(u)},$$

where *U* is a variable that takes values *u* with probabilities $\psi^{U}(u)$ with selection and $\varphi^{U}(u)$ under neutrality. Below, we focus on three variables—genotype frequencies (which describe population states), genotypes, and phenotypes.

For a pair of variables *U*, *V*, statistical dependencies are reflected in their joint and conditional KL divergence, *D*(*U*, *V*) and D(U|V) (see *SI Appendix*, section S1 for the definitions). Both are nonnegative quantities, and they follow the chain rule

[2] D(U,V)=D(U)+D(V|U)=D(V)+D(U|V).

The chain rule allows a comparison of the effects of selection on different variables, as well as on the same variable at different times.

### 2.1. Population-Level Information.

**Fig. 1.**
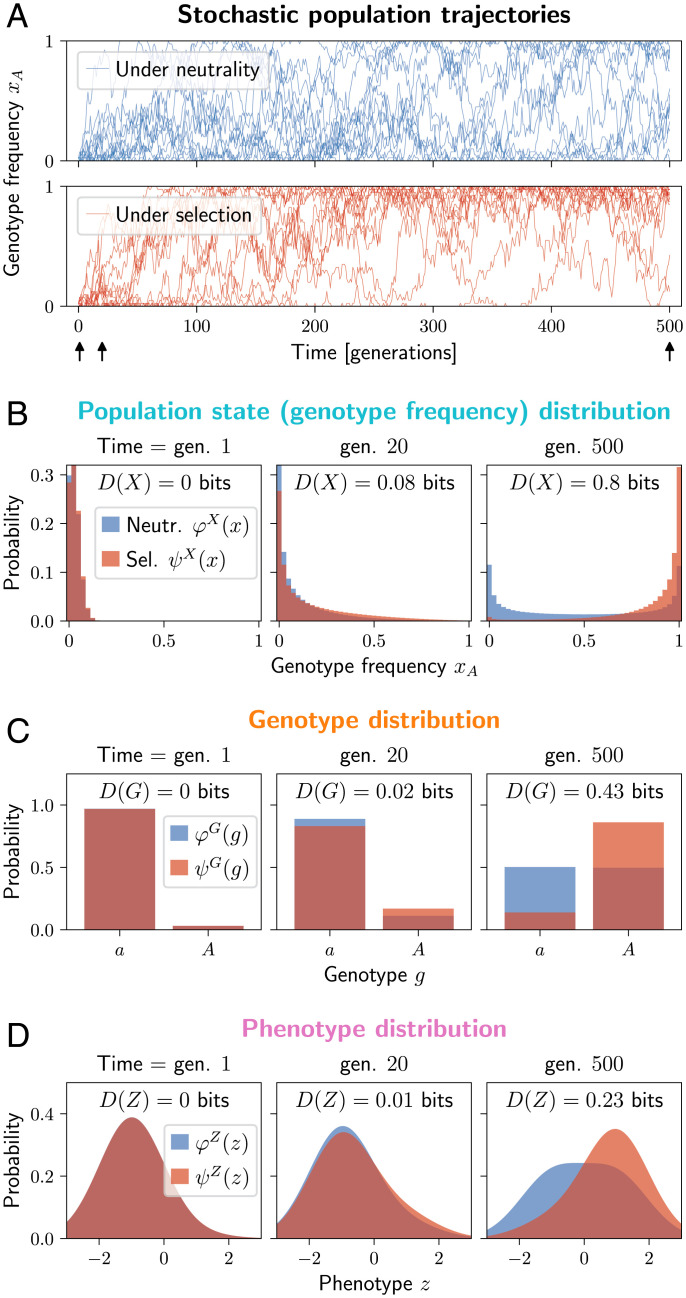
Selection controls the evolution of a single-locus, two-allele system and drives the distribution of the population states, genotype, and phenotype away from neutrality. (*A*) Stochastic trajectories of the frequency *x_A_* of the beneficial allele *A*, under neutrality and under selection (blue and red). The allele *A* starts at a single copy, and, under selection, it tends to increase in frequency. Black arrows indicate the times when the distributions are plotted in *B–D*. At time =500 generations, the system is approximately stationary. (*B–D*) The probability distributions of the genotype frequency *x_A_* (*B*), genotype *g* (*C*), and a noisy phenotype *z* (*D*) under neutrality (blue) and under selection (red) after a varying number of generations of evolution. The associated measures of information *D*(*X*), *D*(*G*), and *D*(*Z*) are indicated. (*B*) The neutral distribution $\varphi^{X}$ converges to a symmetric U shape, while the distribution under selection is biased toward high frequencies of the beneficial allele *A*. The information *D*(*X*) increases over time. (*C*) The neutral genotype distribution $\varphi^{G}$ converges to a uniform distribution, due to symmetry between alleles *a* and *A*. Under selection (*ψ^G^*), the beneficial allele *A* has a higher probability, but it does not dominate completely, so the genotype-level information *D*(*G*) is less than the maximum one bit. *D*(*G*) is also upper bounded by *D*(*X*). (*D*) A phenotype with different means and a Gaussian noise for each allele, $\zeta (z|g)=N(z;\mu_{g},\sigma )$ with $\mu_{a}=-1,\;\mu_{A}=+1$, and σ=1. The information *D*(*Z*) is upper bounded by *D*(*G*), with a gap due to the partially overlapping distributions ζ(z|a) and ζ(z|A). Generated using a haploid Wright–Fisher model (*SI Appendix*, section S4) with population size *N* = 40, mutation rate μ=0.005, and fitness 1 (allele *a*) and 1.05 (allele *A*).

Evolution is a stochastic process happening to populations, and genotype frequencies form the state space. We use *X* to denote the genotype frequencies as a random variable, with each value *x* being a vector with an element *x_g_* for each genotype *g*, normalized as $\sum_{g}x_{g}=1$. As an example, Fig. 1*A* shows a common evolutionary scenario where a single-locus, two-allele system starts from a single copy of a beneficial allele *A*, and, later, the frequency evolves stochastically.

*X* takes values *x* with probabilities $\psi^{X}(x)$ under selection and $\varphi^{X}(x)$ under neutrality. Fig. 1*B* shows examples of these distributions for the single-locus system at three different times. In general, these distributions are shaped by various evolutionary forces—mutation, drift, recombination, selection (*ψ^X^* only), and others. We refer to *D*(*X*), the KL divergence between *ψ^X^* and $\varphi^{X}$, as the population-level information.

The example in Fig. 1 illustrates two important phenomena we discuss in the rest of the paper. The first phenomenon is the accumulation of information. A population evolves from an initial distribution (in the simplest case, $\psi^{X}=\varphi^{X}$ and *D*(*X*) = 0, but this is not necessary). For example, the initial state *x* may be completely specified as in Fig. 1*A*, or both *ψ^X^* and $\varphi^{X}$ may start at the neutral stationary distribution. Over time, selection causes *ψ^X^* to diverge from $\varphi^{X}$, and the information *D*(*X*) accumulates (Fig. 1*B*). We study this in detail in Section 3. The second phenomenon is the maintenance of information, and it takes place when both $\psi^{X}(x)$ and $\varphi^{X}(x)$ are stationary, and the information *D*(*X*) is constant. In Section 4, we study how much information can be maintained at a given cost of selection.

The population-level information *D*(*X*) has been studied under different names and in different roles (7, 34–36). It captures any departure of the genotype frequency distribution *ψ^X^* from its neutral counterpart $\varphi^{X}$—notably, selection can favor not only high frequencies of fit genotypes but also higher or (more typically) lower amounts of genetic variation within populations. Note that *D*(*X*) refers to the effects of selection on the genotype frequencies, rather than allele frequencies. It therefore includes effects of selection on correlations between loci (linkage disequilibrium), which are generated by physical linkage, by chance in finite populations, or due to functional interactions (epistasis)—see also *SI Appendix*, section S2.

Notably, *D*(*X*) (or *D*(*G*) introduced below) appears as a term in free fitness—a quantity analogous to free energy which, under some assumptions, increases over time (35, 37, 38). This implies that evolution maximizes the expected log-fitness while constraining *D*(*X*)—see *SI Appendix*, section S8.

### 2.2. Genotype-Level Information.

If we sample a random genotype from a population in a given state *x*, we find the genotype *g* with a probability given simply by its frequency $\psi^{G|X}(g|x)=\varphi^{G|X}(g|x)=x_{g}$. Taking into account evolutionary stochasticity, we average over all population states *x* with their probabilities $\varphi^{X}(x)$ or $\psi^{X}(x)$,

[3] $$\varphi^{G}(g)=\sum_{x}\varphi^{X}(x)\;x_{g},\;\;\psi^{G}(g)=\sum_{x}\psi^{X}(x)\;x_{g}.$$

Under symmetric point mutations, the neutral distribution $\varphi^{G}$ converges to a uniform distribution over all genotypes, while selection typically concentrates *ψ^G^* among a smaller number of fit genotypes. This is also the case for the single-locus system in Fig. 1*C*. The divergence between *ψ^G^* and $\varphi^{G}$ is the genotype-level information *D*(*G*).

If selection precisely specifies *n* out of *l* nucleotides in the genome—that is, $\psi^{G}(g)$ is uniform over a fraction $1/4^{n}$ out of $4^{l}$ possible genotypes—this implies D(G)=2n bits. This corresponds to the intuition of 2*n* bits of information encoded in the genome. More typically, selection will specify many sites only weakly (biasing the probability toward some alleles; see also Fig. 1*C*), and may contribute to *D*(*G*) through linkage disequilibrium—correlations between linked or epistatically interacting sites. Without linkage or epistasis, *D*(*G*) is approximately additive across loci (*SI Appendix*, Fig. S1).

*D*(*G*) generalizes some previous definitions of genetic information (1, 3, 6) which focused on strong selection or uniform distributions, and coincides with others in important special cases (4, 5).

### 2.3. Phenotype-Level Information.

Finally, selection controls evolution on the level of the phenotype *Z*. *Z* could be a categorical trait such as the presence/absence of a disease or the correct/incorrect protein fold, a quantitative trait, a comprehensive characterization of an individual, or its fitness. Given a genotype *g*, the probability of the phenotype *z* will be given by the possibly noisy genotype–phenotype relationship $\psi^{Z|G}(z|g)=\varphi^{Z|G}(z|g)=\zeta (z|g)$. When there are no environmental effects or intrinsic noise, ζ(z|g) will be concentrated at a single value *z* for each genotype *g*. Taking into account the variation within populations, as well as the evolutionary stochasticity, the marginal probability of *z* is

[4] $$\psi^{Z}(z)=\sum_{g}\psi^{G}(g)\;\zeta (z|g),\;\varphi^{Z}(z)=\sum_{g}\varphi^{G}(g)\;\zeta (z|g).$$

We show the distributions *ψ^Z^*, $\varphi^{Z}$ for the single-locus system in Fig. 1*D*, where the trait has a genotype-dependent mean and Gaussian noise. While, under neutrality, $\varphi^{Z}$ tends to spread out over time, selection causes *ψ^Z^* to be more concentrated. The divergence between *ψ^Z^* and $\varphi^{Z}$ is the phenotype-level information *D*(*Z*).

If we can take the genotype distribution $\varphi^{G}$ to be uniform over all possible DNA sequences of some length, then $\varphi^{Z}$ is the phenotype distribution among such random sequences. Examples of this distribution have recently been measured experimentally for gene expression generated by random promoter sequences in *Saccharomyces cerevisiae* and *Escherichia coli* (26, 28). If a healthy cell requires the gene expression to be in some narrow range, this translates to a requirement on the phenotype-level information *D*(*Z*), and this requirement will increase if the expression needs to be specified across cell states.

### 2.4. The Relationship between the Three Levels.

The definitions above, combined with the chain rule (Eq. **2**) lead to a hierarchy among the three levels,

[5] D(X)≥D(G)≥D(Z).

This inequality can be observed across the columns of Fig. 1*B–D*.

Intuitively, the phenotype-level information *D*(*Z*) is bounded by the genotype-level information *D*(*G*), since the information about the phenotype has to be encoded in the genome. A special case of this relationship has been noted by Worden (3), who, however, worked in a deterministic setting (*SI Appendix*, section S3). The difference between the two, D(G)−D(Z)=D(G|Z), can have two sources. First, the phenotype distribution ζ(z|g) may overlap between genotypes, causing the phenotype to be specified less precisely than the genotype (as in Fig. 1*D*). Second, selection may favor genotypes based on criteria other than the phenotype *Z*, such as other phenotypes or robustness.

**Fig. 2.**
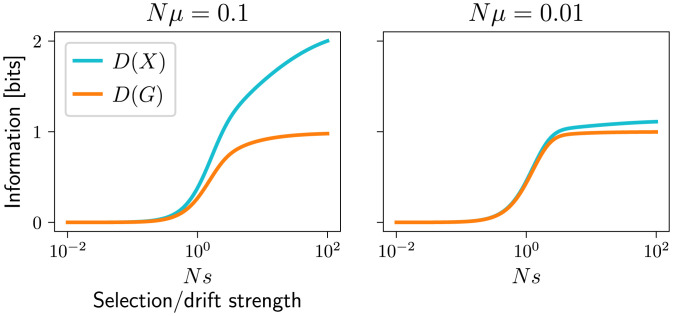
Illustration of *D*(*X*) (cyan) and *D*(*G*) (orange) for a single-locus, two-allele system at stationary distributions $\psi^{X},\varphi^{X}$ as a function of selection strength *Ns* for two different mutation strengths Nμ. The genotype-level information *D*(*G*) grows with *Ns*; from zero up to one bit, when one out of the two alleles dominates, with the steepest increase around *Ns* = 1. The population-level information *D*(*X*) can be much greater than *D*(*G*) when mutation is strong, and generates diversity within the population that selection can shape (or suppress). When mutation is weak, *D*(*X*) and *D*(*G*) are similar, since the population state can be specified by the allele that is currently fixed, and D(X|G)=0. Computed using a Wright–Fisher model as in Fig. 1, with population size *N* = 100.

Similarly, *D*(*G*) can only be as large as the population-level information *D*(*X*). To increase the probability of a genotype *g*, selection must increase the probability of population states with a high frequency of *g*. However, selection can also shape the patterns of genetic diversity in populations, without impacting the average genotype frequencies, therefore contributing to the difference D(X)−D(G)=D(X|G). In populations with weak mutation, which tend to have little diversity, this difference is small—see Fig. 2.

We rely on the inequalities in Eq. **5** in two ways. First, an upper bound on the population-level information *D*(*X*) which we prove in Section 3 also implies an upper bound on the genotype and phenotype-level information *D*(*G*) and *D*(*Z*). In other words, selection can only fine-tune the phenotype to the degree to which it can control the population state.

Second, *D*(*X*) and *D*(*G*) can be difficult to estimate directly for systems with multiple loci, due to the high dimensionality (*SI Appendix*, Fig. S1). In such situations, *D*(*Z*) for fitness or a low-dimensional phenotype *Z* can serve as a lower bound on *D*(*G*) and *D*(*X*). If *Z* is the trait under selection, or fitness itself, this lower bound can be tight. This approach is applicable even for essentially black box genotype–phenotype models, such as models of gene regulation or protein folding.

## 3. Accumulation of Information

In this section, we show how the rate at which *D*(*X*), the population-level information, increases over time is limited by the population size and the variation in fitness. We start by pointing out a connection between population genetics and control theory.

### 3.1. Accumulation of Information and the Cost of Control.

We consider a population evolving over time, with a trajectory $X^{0},X^{1},\ldots ,X^{T}$ forming a Markov chain between generations 0 and *T* (such as in Fig. 1*A*). The divergence of the trajectories’ distribution from neutrality, $D(X^{0},X^{1},\ldots ,X^{T})$, has been proposed as a measure of predictability of evolution (21). Using the chain rule (Eq. **2**), we can decompose it in two ways,

$$D(X^{0},X^{1},\ldots ,X^{T})$$

[6] $$\;=\underset{\overset{\text{Initial}}{\text{information}}}{\underset{︸}{D(X^{0})}}+\underset{\text{KL}\;\text{cost}\;\text{of}\;\text{control}}{\underset{︸}{\sum_{t=0}^{T-1}D(X^{t+1}|X^{t})}}$$

[7] $$\;=\underset{\overset{\text{Final}}{\text{information}}}{\underset{︸}{D(X^{T})}}+\underset{\overset{\text{Effect}\;\text{of}\;\text{selection}\;\text{on}}{\text{trajectories}\;\text{reaching}\;X^{T}}}{\underset{︸}{\sum_{t=0}^{T-1}D(X^{t}|X^{t+1})}}.$$

In Eq. **6**, we distinguish between the divergence of the initial states *X*^0^ and the additional conditional divergence in each generation, $D(X^{t+1}|X^{t})$. The latter can be recognized as the KL cost of control, averaged over the initial states *x^t^* (30, 31). In the context of population genetics, selection takes the role of control.

Eq. **7** makes the distinction between the distribution of endpoints *X^T^*, and the conditional distribution of the states that precede those endpoints. Selection can shape the full trajectories, but only the effects on *X^T^* constitute the final population-level information.

Together, Eqs. **6** and **7** imply a bound on the information accumulated between times 0 and *T* in terms of the KL cost of control,

[8] $$D(X^{T})-D(X^{0})\leq \sum_{t=0}^{T-1}D(X^{t+1}|X^{t}).$$

Specifically, the information accumulated over a single generation, $\Delta D(X^{t})=D(X^{t+1})-D(X^{t})$, is upper bounded as

[9] $$\Delta D(X^{t})\leq D(X^{t+1}|X^{t}).$$

Analogous bounds for continuous time Markov chains and the diffusion approximation are provided in *SI Appendix*, sections S6 and S7.

Note that control theory is concerned with computing optimal control policies, which maximize an imposed objective while minimizing the cost $\sum_{t=0}^{T-1}D(X^{t+1}|X^{t})$. This is analogous to computing the optimal artificial selection—in fact, the KL divergence control theory framework has recently been used to study artificial selection on quantitative traits (32).

In contrast, natural selection is typically given by the biological or ecological circumstances, and not necessarily optimized in this sense. Still, the KL cost of control provides bounds on the rate at which selection accumulates information (Eqs. **8** and **9**), and it has a meaning in population genetics, which we discuss in the next section.

We also note that Eq. **9** is related to the proof that free fitness increases over time (37, 38); see *SI Appendix*, section S8.

### 3.2. Variation in Fitness as Cost of Control.

To compute $D(X^{t+1}|X^{t})$ in population genetics, we need to specify a model. We analyze multiple general model classes in *SI Appendix*: Wright–Fisher and discrete Moran models in *SI Appendix*, section S5, continuous time Moran model in *SI Appendix*, section S6, and the diffusion approximation in *SI Appendix*, section S7. In summary, the bound in Eq. **9** always takes the form

[10] $$\Delta D(X^{t})\leq kN\sum_{x^{t}}\psi^{X^{t}}(x^{t})\;C(x^{t})=kN{\langle C\rangle }_{t},$$

where *N* is the population size, *kN* is the number of individuals that are sampled with selection in each generation (*k* = 1 under asexual reproduction and *k* = 2 under sexual reproduction when two parents are sampled with selection for each individual). $C(x^{t})$ is the cost of selection at the population state *x^t^* (see below), and ${\langle C\rangle }_{t}$ is the expected cost at time *t*. To upper bound information accumulated over multiple generations, we need to sum over them,

[11] $$D(X^{T})-D(X^{0})\leq kN\sum_{t=0}^{T-1}\sum_{x^{t}}\psi^{X^{t}}(x^{t})\;C(x^{t})=kN{\langle C\rangle }_{0,T}.$$

The cost *C*(*x*) is a measure of fitness variation in a population in the state *x*,

[12] $$C(x)=\sum_{g}x_{g}{\hat{w}}_{g}(x)\log_{2}{\hat{w}}_{g}(x),$$

where ${\hat{w}}_{g}(x)$ is the (frequency dependent) relative fitness of genotype *g*. When sampling genotypes as parents for the next generation, *g* is picked with probability *x_g_* under neutrality and $x_{g}{\hat{w}}_{g}(x)$ under selection—*C*(*x*) is the KL divergence between these two distributions.

*C*(*x*) is related to two more established measures of cost in population genetics—the relative fitness variance *V*(*x*) and the genetic load *L*(*x*), which have been studied under a number of circumstances—for example, mutation–selection balance (39), genetic drift (40, 41), certain types of epistasis and the evolution of sex (42, 43), ongoing substitutions (44–46), or stabilizing selection on quantitative traits (47). They are defined as

[13] $$V(x)=\sum_{g}x_{g}{\left({\hat{w}}_{g}(x)-1\right)}^{2}$$

[14] $$L(x)=1-\frac{1}{{\hat{w}}_{\text{max}}(x)},$$

where ${\hat{w}}_{\text{max}}(x)$ is the maximum relative fitness present in the population *x*, ${\hat{w}}_{\text{max}}(x)=\max_{g;\;x_{g}>0}\;{\hat{w}}_{g}(x)$. We derive the relationships between *C*(*x*), *V*(*x*), and *L*(*x*) in *SI Appendix*, section S9. *V*(*x*) and *L*(*x*) satisfy the inequality V(x)≤[L(x)]/[1−L(x)] (see also ref. 48), and both provide an upper bound on *C*(*x*),

[15] $$C(x)\leq \frac{V(x)}{\ln2},\;\;C(x)\leq \log_{2}\frac{1}{1-L(x)}.$$

In addition, under weak selection and in the diffusion approximation, C(x)=V(x)/(2log2). The bounds in Eqs. **10** and **11**. can therefore also be rewritten in terms of *V*(*x*) or *L*(*x*) using Eq. **15**.

Assuming constant population size, relative fitness is proportional to the expected number of offspring, and therefore limited by the species’ reproductive capacity. The quantities ${\hat{w}}_{\text{max}}(x)$, *L*(*x*), *V*(*x*), and *C*(*x*), and, as a consequence, ΔD(X), are therefore all limited in realistic settings (*SI Appendix*, section S9).

In the context of artificial selection or genetic algorithms, an alternative measure of cost is the population size *N*, which is the number of cultivated plants or animals, or fitness function evaluations (49, 50). We note that, according to the bounds in Eqs. **10** and **11**, the maximal accumulation rate is also proportional to *N*. Furthermore, increasing the strength of selection (and therefore *C*(*x*)) beyond an optimal value may increase the immediate response to selection, but it reduces the long-term response, due to loss of genetic diversity (49, 50). Therefore, in practice, *C*(*x*) will be limited even in this context.

### 3.3. Example 1: The Fates of a Beneficial Allele.

The bounds in Eqs. **10** and **11** hold in genetically diverse populations with clonal interference or recombination. Still, it is interesting to consider the case of sequential fixation/loss of mutations, as was done previously (1, 7, 44).

**Fig. 3.**
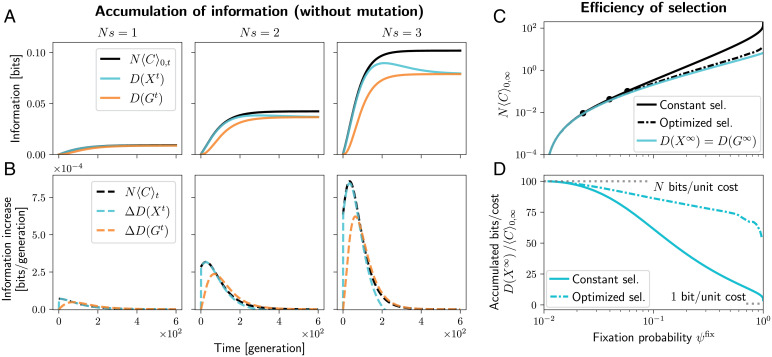
Information accumulation associated with the fixation or loss of a beneficial allele in a haploid single-locus, two-allele system. The beneficial allele starts at a single copy and evolves under drift and selection, but no mutation. (*A*) The population-level information ($D(X^{t})$, cyan) and genotype-level information ($D(G^{t})$, orange) over time, for three different strengths of selection: *Ns* = 1 (*Left*), *Ns* = 2 (*Middle*) and *Ns* = 3 (*Right*). Both $D(X^{t})$ and $D(G^{t})$ start at zero, accumulate over time as selection tends to increase the frequency of the beneficial allele, and saturate as the allele is fixed or lost. The black line is the upper bound according to Eq. **11** with *k* = 1. (*B*) The increments in $D(X^{t})$ and $D(G^{t})$ per generation (cyan and orange dashed lines), and the upper bound according to Eq. **10** with *k* = 1 (black dashed line). (*C*) The cyan line shows the total information accumulated, $D(X^{\infty })=D(G^{\infty })$, as a function of the fixation probability $\psi^{\text{fix}}$. $D(X^{\infty })$ serves as a lower bound on *N* times the total cost of selection, plotted in black, regardless of the form selection takes. The full black line corresponds to constant selection coefficient, with black points showing the three cases in *A* and *B*. The dash-dotted black line shows frequency-dependent selection that maximizes $\psi^{\text{fix}}$ (and therefore also $D(X^{\infty })$) while constraining $N{\langle C\rangle }_{0,\infty }$. (D) Same data as in C, but the vertical axis now shows the ratio of the information $D(X^{\infty })$ and the total cost of selection ${\langle C\rangle }_{0,\infty }$ for constant selection (full black) and optimized frequency-dependent selection (dash-dotted black line). At most, *N* bits can be accumulated per unit cost, and this is achieved at weak selection. At strong selection, this reduces to as low as one bit per unit cost. Figure computed using the Wright–Fisher model as in Fig. 1, with population size *N* = 100.

Suppose that a beneficial allele *A* appears in one copy at time *t* = 0, and is guaranteed to be fixed or lost before another mutation appears that could interfere with it. The population and genotype-level information, $D(X^{t})$ and $D(G^{t})$, start at zero and accumulate over time, as selection tends to increase the frequency of *A* (Fig. 3*A*). The cumulative cost of selection $N{\langle C\rangle }_{0,t}$ serves as the upper bound on both $D(X^{t})$ and $D(G^{t})$.

Note that, under relatively strong selection (*Ns* = 3; Fig. 3*A*, *Right*), *A* increases in frequency considerably faster than under neutrality, leading to high $D(X^{t})$. But some of these gains are later lost as *A* is fixed or lost. This is an example of how only the probabilities of endpoints, and not the shape of the trajectories, matters for the information that is ultimately accumulated (the two terms in Eq. **7**).

The increments in $D(X^{t})$ and $D(G^{t})$ in each generation are plotted in Fig. 3*B*, along with the bound by $N{\langle C\rangle }_{t}$, Eq. **10**. The bound on $\Delta D(X^{t})$ is relatively tight. $\Delta D(G^{t})$ can temporarily exceed $N{\langle C\rangle }_{t}$, since the accumulation bound in Eq. **10** does not directly apply to the genotype level, but this is only a transient phenomenon due to the inequality between the cumulative genotype- and population-level information $D(G^{t})\leq D(X^{t})$.

Both $D(X^{t})$ and $D(G^{t})$ saturate at the same value $D(X^{\infty })=D(G^{\infty })$, since the ultimate fate of the population is given simply by whether the allele *A* is fixed or lost. The fixation probability is 1/N under neutrality and $\psi^{\text{fix}}=\psi^{X^{\infty }}((1))=\psi^{G^{\infty }}(A)$ under selection, and the accumulated information is a function of this probability,

[16] $$D(X^{\infty })=D(G^{\infty })=$$

[17] $$\;=\psi^{\text{fix}}\log_{2}(N\psi^{\text{fix}})+(1-\psi^{\text{fix}})\log_{2}\frac{N(1-\psi^{\text{fix}})}{N-1}.$$

This function is plotted in cyan in Fig. 3*C*. According to Eq. **11**, it provides a lower bound on the total cost, $N{\langle C\rangle }_{0,\infty }\geq D(X^{\infty })$, given a fixation probability. This holds when the allele *A* has a constant, frequency-independent selective advantage, as in the three examples in Fig. 3*A* and *B* (full black line and black points in Fig. 3*C*). By computing a suitable frequency-dependent selection, which optimizes the fixation probability while constraining the total cost $N{\langle C\rangle }_{0,\infty }$, we can reduce the cost considerably (dash-dotted black line in Fig. 3*C*; see *SI Appendix*, section S11 and Fig. S4 for details). This is achieved by making selection weaker at high frequencies, where the risk of losing *A* is low. Still, the cost stays above $D(X^{\infty })$, as it has to under arbitrary frequency and time-dependent selection.

Under both forms of selection, the bound is only tight when selection is weak. To emphasize this, we plot the information accumulated per unit cost, $D(X^{\infty })/{\langle C\rangle }_{0,\infty }$, as function of the fixation probability $\psi^{\text{fix}}$ in Fig. 3*D*. At weak selection, $\psi^{\text{fix}}$ is only perturbed a little from its neutral value 1/N, but up to *N* bits can be accumulated per unit cost. A special case of this was shown by Barton (7). Similar scaling with *N* was also found in a different setting by Kimura (45).

Stronger selection accumulates more information, but at a disproportionately higher cost, since a large part of it is spent on shaping trajectories rather than outcomes. In the extreme case, to achieve $\psi^{\text{fix}}=1$, only individuals carrying the *A* allele can be allowed to reproduce, and *A* gets fixed in only one generation—a highly unlikely way to fixation under neutrality. In this case, selection has the same effect on each genotype sampled as a parent in the first generation as it does on the allele that is ultimately fixed (both are *A* with probability 1/N under neutrality and one under selection). As a result, the cost is equal to the accumulated information, ${\langle C\rangle }_{0,\infty }=D(G^{\infty })=D(X^{\infty })$, and only one bit per unit cost is accumulated (Fig. 3*D*). This is why previous results derived in deterministic settings (1, 3) claimed much more stringent limits on accumulation of information.

### 3.4. Example 2: Accumulation of Information under Mutation.

Unlike the example above, real systems experience ongoing mutation. On the one hand, mutation is necessary to supply beneficial alleles for adaptation, but, on the other hand, mutation can disrupt existing adaptation. In this section, we assume that the single-locus, two-allele system starts at the neutral stationary distribution with $D(X^{0})=D(G^{0})=0$, and then selection is turned on. Adaptation exploits copies of the allele *A* that either segregate in the population by chance at time 0, or arise later by mutation.

**Fig. 4.**
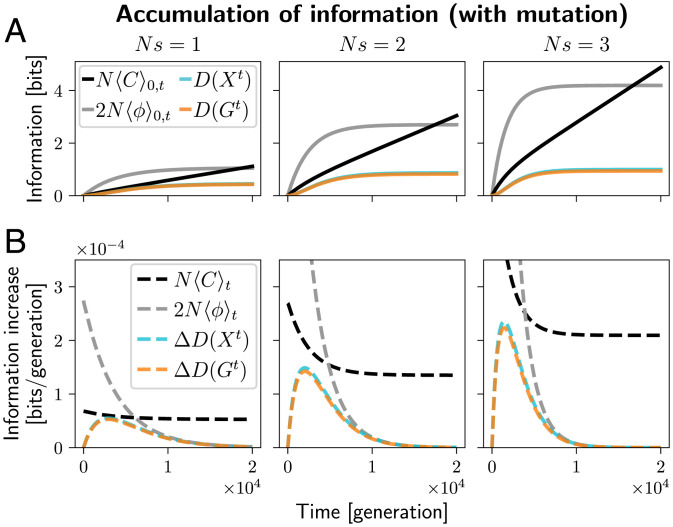
Information accumulation in a single-locus, two-allele system and the associated upper bounds. The system starts from a neutral stationary distribution over allele frequencies, where $D(X^{0})=D(G^{0})=0$. Then it evolves under selection with varying strengths (*Ns* = 1 (*Left*), *Ns* = 2 (*Middle*) and *Ns* = 3 (*Right*)) for $2\times 10^{4}$ generations. (*A*) The cumulative information at the population level ($D(X^{t})$, cyan) and genotype level ($D(G^{t})$, orange) over time. Due to the weak mutation Nμ=0.01, the two measures of information are similar. The black and gray lines show upper bounds by the cumulative cost of selection and the cumulative fitness flux. (*B*) The increments in information per generation, $\Delta D(X^{t})$ (cyan dashed line) and $\Delta D(G^{t})$ (orange dashed line) and the upper bounds on these increments in terms of the cost of selection $kN{\langle C\rangle }_{t}$ (black, in this case *k* = 1) and the expected fitness flux $2N{\langle \psi \rangle }_{t}$ (gray) *Ns* = 1 (*Left*), *Ns* = 2 (*Middle*) and *Ns* = 3 (*Right*). Note that the cost of selection bound is briefly nearly tight under weak selection (*Ns* = 1, *Left*), and the fitness flux bound is tight near stationarity, when both the accumulation rate and the fitness flux approach zero. Figure computed using the Wright–Fisher model as in Fig. 1. The population size is fixed at *N* = 100. For technical reasons, the expected fitness flux curves were computed using an equivalent Moran model; see *SI Appendix*, section S10 and Fig. S2.

Fig. 4*A* shows the information $D(X^{t})$ and $D(G^{t})$ over time. Accumulation take place on the time scale of 1/μ. Note that the bound Eq. **11** is not very tight. This is even more apparent in Fig. 4*B*, where the average cost per generation $N{\langle C\rangle }_{t}$ remains positive even after the system has reached the new stationary state, while the increments in $D(X^{t})$ and $D(G^{t})$ are zero. This corresponds to the cost of maintaining information, which we discuss in Section 4.

In summary, the accumulation of information is upper bounded by the KL cost of control, which, in turn, corresponds to the population size times the variation in fitness. However, if selection changes not only the probabilities of the final states but also the paths that lead there (because it is strong, because adaptation is maintained for a long time, or because adaptation is reversed by time-dependent selection), then the information accumulated is less than the total cost.

### 3.5. Comparison with the Fitness Flux Bound.

The fitness flux theorem (35) implies another upper bound on information accumulation rate, $\Delta D(X^{t})\leq 2N{\langle \phi \rangle }_{t}$, where ${\langle \phi \rangle }_{t}$ is the expected fitness flux per generation. It is plotted in gray in Fig. 4. It differs from the cost of selection bound both quantitatively and in terms of interpretation.

Quantitatively, neither bound is tighter in general. In Fig. 4*B*, the cost of selection bound is tighter in early stages of adaptation, and the fitness flux bound is tighter in the late stages. This is consistent with the interpretation of fitness flux as the rate of ongoing adaptation, or the rate of ascent in the mean fitness landscape/seascape (35). This rate is high in the early stages of adaptation, when the population is far from the fitness peak and tends to climb up quickly. Later, when the population approaches a stationary distribution, there is no more adaptation, on average, and $2N{\langle \phi \rangle }_{t}$ as well as $\Delta D(X^{t})$ vanish. Meanwhile, the cost of selection bound $kN{\langle C\rangle }_{t}$ is tighter in the earlier stages when most of the cost is spent on new adaptation, but it remains positive under stationarity, due to maintenance costs.

Technically, the fitness flux theorem was originally derived in ref. 35 under the diffusion approximation, and requires an additional assumption that the neutral process is at a stationary distribution with detailed balance. We derive and discuss the technical aspects of the fitness flux bound in *SI Appendix*, section S10 and Figs. S2 and S3.

## 4. Maintenance of Information

In this section, we ask how much information can be maintained in the genome for a given cost of selection. A general bound analogous to Eq. **10** seems to be out of reach for now, but we can study how the information maintained depends on key evolutionary parameters. We start by analyzing the single-locus, two-allele system, and then proceed to systems with large numbers of loci.

### 4.1. Single Locus: Weak Selection Is Most Efficient.

**Fig. 5.**
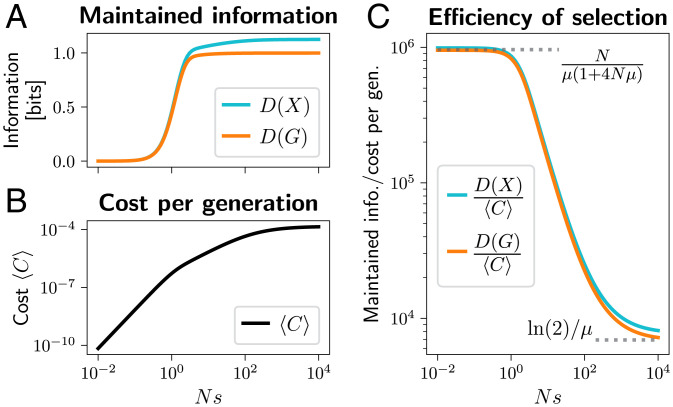
Maintenance of information in the single-locus, two-allele system. (*A*) The stationary values of information, *D*(*X*) (cyan) and *D*(*G*) (orange), as function of selection strength *Ns*. Stronger selection keeps the beneficial allele at higher frequencies, but this is associated with higher average cost of selection 〈C〉, shown in *B*. Note that, much of the time, one of the alleles is fixed, and the cost *C* is zero. 〈C〉 is the average cost per generation over the stationary distribution of allele frequencies. (*C*) The ratio of the maintained information and the average cost of selection, D(X)/〈C〉 (cyan) and D(G)/〈C〉 (orange). Selection is most efficient when it is relatively weak (Ns≪1), maintaining up to N/μ(1+4Nμ) bits per unit cost at the genotype level, and inefficient when strong (Ns≫1), maintaining only about ln(2)/μ bits per unit cost (dotted horizontal lines). The population size is *N* = 100, and the mutation rate is $\mu =10^{-4}$.

Fig. 5*A* shows the information, *D*(*X*) and *D*(*G*), maintained by the single-locus, two-allele system at the stationary state under various strengths of selection. Stronger selection maintains more information—up to one bit at the genotype level, and more on the population level. However, it comes with a higher cost of selection 〈C〉 (Fig. 5*B*). Notably, the cost increases faster than the maintained information. As a result, the amount of information maintained per unit cost decreases with selection strength (Fig. 5*C*).

There are two important asymptotic regimes. When selection is very strong, Ns≫1, deleterious mutations are purged as soon as they arise, and D(G)≈1 bit. Mutations arise with a probability Nμ per generation, and purging each costs C≈1/(Nln(2)) (assuming truncation selection with α=1−1/N; see *SI Appendix*, section S9). In this regime,

[18] $$\text{Strong}\;\text{selection}:\;\;\frac{D(G)}{\langle C\rangle }\approx \frac{\ln2}{\mu },$$

bits can be maintained per unit cost (Fig. 5*C*). Similar arguments apply when Nμ>1. The inverse scaling with *μ* is expected based on the deterministic mutation load (39) or Eigen’s error catastrophe (2) which occurs when selection cannot maintain sequences without error, and it was also derived by Watkins (5).

Selection is much more efficient when it is weak, Ns≪1. Both the cost and the maintained information can be calculated under the diffusion approximation (see *SI Appendix*, section S4B for details). If mutation is also weak, Nμ≪1, the amount of genetic variation (pairwise diversity) scales with 2Nμ, and the cost (variation in fitness) is approximately $\langle C\rangle \approx N\mu s^{2}/(2\ln2)$. Meanwhile, selection shifts the mean frequency of *A* away from 1/2 by about Ns/2, and this is associated with genotype-level information $D(G)\approx N^{2}s^{2}/(2\ln2)$ bits. In this regime, up to N/μ bits per unit cost are maintained. When mutation Nμ is not negligible, a more accurate result is

[19] $$\text{Weak}\;\text{selection}:\;\;\frac{D(G)}{\langle C\rangle }\approx \frac{N}{\mu (1+4N\mu )};$$

see *SI Appendix*, section S4. This limit is also highlighted in Fig. 5*C*. The special case when $N\mu \gg 1,\;D(G)/\langle C\rangle \approx 1/(4\mu^{2})$, was previously derived by Watkins (5).

By itself, a single locus under weak selection cannot contribute much to biological function. However, selection can act on a polygenic trait influenced by many loci. If they are unlinked, we expect both the maintained information and the cost of selection to be approximately additive, and the ratio D(G)/〈C〉 to scale according to Eq. **19**. To confirm this, we next study a polygenic system.

### 4.2. Information Stored among Many Loci.

We use an individual-based model to study a population of *N* haploids with *l* = 1, 000 biallelic loci, mutation and free recombination. Offspring are produced by sampling pairs of parents with selection, shuffling their genomes (at each locus, the allele from either parent is inherited with probability 1/2), and flipping each allele with probability *μ*. Selection acts on a fully heritable, additive trait with equal effects, $z_{g}=(\text{the}\;\text{number}\;\text{of}\;A\;\text{alleles}\;\text{in}\;g)$, with fitness being $w_{g}={\left(1+s\right)}^{z_{g}}$.

**Fig. 6.**
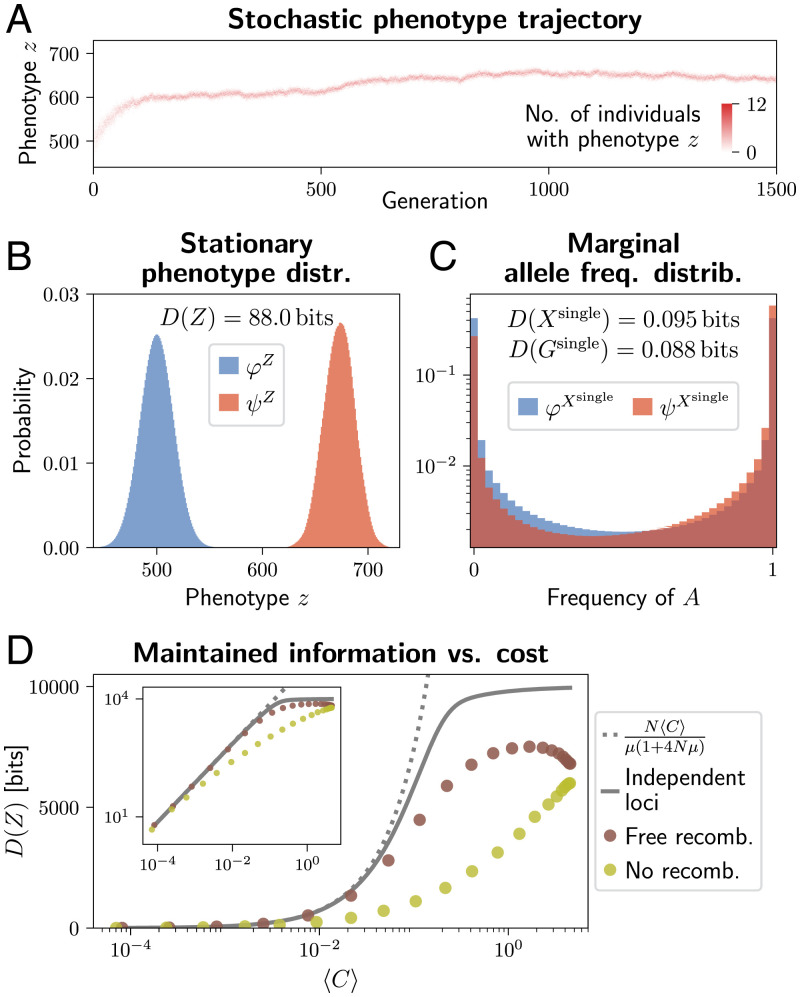
Maintenance of information in a system with *l* = 1, 000 biallelic loci. Selection is directional on an additive trait *Z* (= the number of beneficial alleles). (*A*) A heatmap showing the number of individuals in a population occupying each value of the phenotype *z* at each generation. The population is initialized as a collection of random genomes, each containing the beneficial allele at around l/2=500 loci. Over time, this number stochastically increases due to selection. Only the first 1,500 generations of the trajectory are shown; the full trajectory was $5\times 10^{3}$ generations of burn-in and $2\times 10^{5}$ to estimate the stationary distributions in *B* and *C*. (*B*) The stationary distribution over the phenotype *Z*, under neutrality ($\varphi^{Z}$, blue) and selection (*ψ^Z^*, red), along with the phenotype-level information *D*(*Z*). Due to symmetry between loci and alleles, $\varphi^{Z}(z)=\text{Binom}(z;l,0.5)$ is binomial. Under selection, *ψ^Z^* is obtained as the histogram over individuals and over $2\times 10^{5}$ generations at stationarity. (*C*) The marginal distribution over allele frequencies at individual loci, under neutrality ($\varphi^{X^{\text{single}}}$, blue, computed using a transition matrix for the single-locus system) and under selection ($\psi^{X^{\text{single}}}$, red, computed as a histogram over all loci and $2\times 10^{5}$ generations at stationarity). The associated $D(X^{\text{single}})$ and $D(G^{\text{single}})$ correspond to information maintained at one locus, and, because the loci are approximately independent, the total information is about l=1,000 times more. The population size is *N* = 40, the mutation strength is Nμ=0.02, and the selection strength is *Ns* = 0.4. (*D*) The relationship between the maintained information *D*(*Z*) and the cost of selection 〈C〉, with recombination (brown points) and without recombination (olive points). This is compared with predictions under the assumption of independent loci (gray line; computed using single-locus diffusion approximation and multiplying both information and cost by the number of loci) and the linear scaling with 〈C〉 based on Eq. **19** (dotted gray line). Computed for a system with $l=10^{4}$ loci, population size *N* = 40, mutation strength Nμ=0.02, and variable *Ns*. Distributions estimated from a stochastic trajectory over $5\times 10^{4}$ generations, after $5\times 10^{3}$ generations of burn-in. *Inset* shows identical data with a log vertical scale.

The results are shown in Fig. 6. Fig. 6*A* shows an example of a stochastic population trajectory, indicating the phenotypes present in the population over time. The system is initialized with random genomes that contain the beneficial allele at each locus with probability 1/2, with *z* taking values around l/2=500 with binomial noise. Selection with *s* = 0.01 makes the beneficial alleles more frequent over time. The stationary distribution over phenotypes is shown in Fig. 6*B*. Under neutrality, $\varphi^{Z}=\text{Binom}(l,1/2)$ by symmetry. The distribution *ψ^Z^* under selection is shifted relatively far from $\varphi^{Z}$, leading to D(Z)=88.0 bits of information on the phenotype level.

The population state distribution and the genotype distribution are inaccessible due to their dimensionality (*SI Appendix*, Fig. S1). However, we know that they are lower bounded by *D*(*Z*), which is easy to compute, and D(Z)≈D(G), since *Z* is the only trait under selection. Since the loci are unlinked and have equal effects, the information *D*(*Z*) can be divided evenly among them. The marginal distribution over allele frequencies is only slightly different from neutrality (Fig. 6*C*), by about $D(X^{\text{single}})=0.095$ bits in terms of allele frequency distribution and $D(G^{\text{single}})=0.088$ in terms of allele probabilities. The 1,000 loci, however, combine to produce a large shift in the phenotype distribution, $D(Z)\approx 1,000D(G^{\text{single}})$.

This information is maintained at a very low cost of selection, 〈C〉=0.0012 bits per generation, or relative fitness variance 〈V〉=0.0017. This amounts to $D(Z)/\langle C\rangle =7.1\times 10^{4}$ bits per unit cost, only a little below the single-locus limit $N/\mu /(1+4N\mu )=7.4\times 10^{4}$ under weak selection.

### 4.3. Interference between Loci.

In practice, the selection on different loci might interfere, and this can hinder the maintenance of information. The interaction may be due to Hill–Robertson interference, linkage, or epistasis.

In Fig. 6*D*, we vary the selection coefficient *s* on individual alleles in an $l=10^{4}$ locus system, and plot the maintained *D*(*Z*) against the cost 〈C〉. We use the individual-based model to compute these with free recombination (as in Fig. 6*A*–*C*) and with zero recombination (offspring genotypes are identical to those of single parents, up to mutation). We compare the results with the weak selection scaling according to Eq. **19**, and results for 10^4^ loci that evolve independently (cost and information are summed over 10^4^ single-locus systems).

With free recombination, weak selection maintains about as much information as if the loci were independent (brown points and gray line in Fig. 6*D*, *Inset*), approximately according to Eq. **19** (gray dotted line). However, when selection is strong (〈C〉≈0.1 or more), individual alleles experience additional fluctuations in frequency, due to random associations with alleles at other loci in a finite population (51, 52), reducing the efficiency of selection. As a result, the freely recombining loci maintain less information than if they were independent. This is in addition to the fact that, under strong selection, maintenance is more costly even for independent loci (full gray line departs from dotted gray line, Fig. 6*D*). Extremely strong selection, which removes potentially adaptive variation at other loci, maintains even less information than more moderate selection, and it makes recombination ineffective (brown points at high 〈C〉 in Fig. 6*D*).

Without recombination, less information is maintained at any given cost (olive points in Fig. 6*D*). In fact, Watkins (5) has shown that, due to clonal interference, organisms with no recombination cannot maintain more than the order of ln(N)/μ bits of information even if the cost is unlimited, making Haldane’s (39) and Eigen’s results (2) pertinent to asexual populations.

The advantage of recombination has also been recognized in a similar context by MacKay (4) and Peck and Waxman (6), and relates to the evolution of sex and epistasis. Recombination is advantageous when facing unconditionally deleterious or beneficial alleles (43), but can be disadvantageous when adaptation depends on beneficial combinations of alleles (53). However, it is not clear whether any form of selection can maintain more information at a given cost than N/[μ(1+4Nμ)] achieved by weak directional selection with recombination.

## 5. Discussion

Selection exerts control on evolving populations, but its capacity is limited. The limits to selection have been approached from various angles. Here we build upon previous work that had developed the idea that selection accumulates and maintains information in the genome (1, 2), and that this is associated with a cost in terms of variation in fitness, such as genetic load or fitness variance (39, 44). The early work has suggested remarkably simple limits to selection: that the maximal rate of accumulation is bounded by the cost itself (1, 3), and that maintenance is limited to about 1/μ functional sites in the genome (2, 39).

Later work has pointed out that both accumulation (4, 7) and maintenance (5, 6) can exceed these limits, notably when recombination is involved. However, the general bounds remained unclear, possibly, in part, due to the difficulty of defining genetic information in general.

The measures of information that we have introduced in Section 2 coincide with or generalize previous definitions, and offer two advantages. First, they facilitate connections between different levels—for example, between the abstract population-level information that has been studied theoretically in different contexts (34–36) and the effect that selection has on the distribution of phenotypes.

Second, the generality of our definition allows proving a general bound on information accumulation rate. This turns out to be a factor *N* faster than the early bounds, but depends on selection on individual loci being weak. The bound relies on a measure of cost of selection that connects the genetic load and fitness variance (48) with the KL cost in control theory (30, 31), recently used in the context of artificial selection (32).

How much information can be maintained in the genome at a given cost remains an open problem, but we have discussed how this might scale with the population size and the mutation rate. The scaling in Eq. **19** generalizes a result by Watkins (5) for realistic populations with Nμ<1. Still, more work is needed to make claims about the information content of any real organism’s genome. Typical populations have $N_{e}/\mu$ much greater than the genome size, suggesting that the genome size or other factors are more limiting than Eq. **19**. The maintenance can be made more difficult by linkage or epistasis, and parts of the genome are likely under strong selection which is more costly. Still, Eq. **19** suggests that, in theory, the genome could contain a substantial amount of information among weakly selected loci, for example, coding for polygenic traits. This is consistent with recent work (54) pointing out that mutation load does not pose severe limitations to the functional fraction of the human genome.

Similarly, the bound on accumulation rate in Eq. **10** hypothetically allows accumulation of information amounting to 10% of the human genome in about 10^6^ generations ($6\times 10^{8}$ bits, assuming effective population size $N_{e}\approx 10^{4}$, *k* = 2, and meager cost 〈C〉≈0.03 or relative fitness variance 〈V〉≈0.018 devoted to accumulation). But this is unlikely to have happened. Some selection was likely strong and more costly, and selection could have fluctuated, reversing previous adaptation. However, under the right conditions, information can accumulate very fast.

Our findings are complementary to the point raised by Kondrashov (41), that the survival of populations could be threatened by large numbers of weakly deleterious mutations (*Ns* < 1). While selection cannot purge them, it can perturb the allele frequency distribution of each by a small amount, and thus shift the distribution of higher-level traits very far from neutrality. This is similar to the resolution by Charlesworth (55). In fact, information accumulation and maintenance are most cost efficient in this regime. This does not mean that a genomic architecture, where most mutations operate at *Ns* < 1 and information is encoded among many weakly specified sites, would evolve as an adaptation to maximize information gain. Nevertheless, such an architecture might arise in multicellular organisms as a side effect of their small effective population sizes and long genomes (56, 57).

Focus on the information content of genomes, rather than their fraction under selection, could help better frame the controversy sparked by some publications from the Encyclopedia of DNA Elements (ENCODE) project (12–16, 54, 58). On the one hand, genomic regions under detectable selection [less than 15% in humans (59)] likely contain less than two bits per base pair, because their current function could be achieved by a number of alternative sequences (e.g., due to synonymous mutations in coding regions, or flexibility of transcription factor binding site sequence and location). On the other hand, regions without detectable selection could contain a considerable amount of information in the aggregate, at a low cost, encoding polygenic traits.

In bioinformatics, there already is a measure of information content applicable to short regulatory motifs (18, 19). Future work could examine the precise relationship between this measure and our theoretical definitions. The generality of our framework also opens directions for future research. One is to predict the maximal amount of information that can be maintained in genomes and populations with realistic parameters. Another is to study the information content of genomic elements with well-described genotype–phenotype maps [e.g., promoters (26, 27)], under different hypotheses about selection on the phenotype.

## Supplementary Material

Supplementary File

## Data Availability

There are no data underlying this work.
