# Supplementary material for: Accumulation and maintenance of information in evolution
Source: Proc Natl Acad Sci U S A. 2022 Aug 29;119(36):e2123152119. doi: 10.1073/pnas.2123152119 (PMC9457054; doi:10.1073/pnas.2123152119)
Supplement: Supplementary File [file pnas.2123152119.sapp.pdf]

PNAS
www.pnas.org

Supplementary Information for

Accumulation and maintenance of information in evolution

Michal Hledík, Nick Barton, Gašper Tkačik

Michal Hledík
E-mail: michal.hledik@ist.ac.at

This PDF file includes:

Supplementary text
Figs. S1 to S4 (not allowed for Brief Reports)
SI References

Supporting Information Text

Contents

S1 Joint and conditional KL divergence, chain rule 2
S2 Population-level information: allele frequencies and LD 3
S3 Violation of the bound by Worden 1995 by drift 3
S4 The single locus, two allele system used for figures 3
S4A Wright-Fisher model . . . 4
S4B The diffusion approximation . . . 4
S5 The bound on information accumulation rate – Markov chains 5
S5A Wright-Fisher model . . . 5
S5A.1 Selection in an infinite offspring pool . . . 6
S5A.2 Selection among parents . . . 7
S5B Discrete-time Moran model . . . 7
S6 The bound on information accumulation rate – continuous-time Markov chains 8
S6A Continuous-time Moran model . . . 8
S7 The bound on information accumulation rate – diffusion approximation 9
S7A Application to population genetics . . . 10
S8 Relationship with free fitness and statistical physics 10
S8A Boltzmann form of stationary distributions . . . 10
S8B Free fitness . . . 11
S8C Monotonic convergence of stochastic processes to their stationary distributions . . . 12
S8D Free fitness as a trade-off between fitness and information . . . 12
S9 Properties of measures of cost of selection 13
S10 Fitness flux theorem 14
S10A Discrete-time Markov chains . . . 14
S10B Diffusion approximation . . . 15
S10C Comparisons of the discrete and the diffusion formulas . . . 15
S10D Interpretation of the bounds under diffusion . . . 16
S11 Frequency dependent selection that maximizes fixation probability 17

List of Figures

S1 Distributions on the three levels with multiple loci . . . 18
S2 Fitness flux formulas comparison . . . 19
S3 Bounds from an intermediate frequency . . . 20
S4 Frequency dependent selection . . . 21

S1. Joint and conditional KL divergence, chain rule

For a single variable U, the KL divergence (1) between its distributions with and without selection is

$$D(U) = \sum_u \psi^U(u) \log_2 \frac{\psi^U(u)}{\varphi^U(u)} \quad [S1]$$

where U takes values u with probabilities $\psi^U(u)$ under selection and $\varphi^U(u)$ under neutrality.

To be well defined, the KL divergence requires that the support of ψ^U is a subset of the support of φ^U. In other words, if for some u we have $\varphi^U(u) = 0$, then also $\psi^U(u) = 0$ – outcomes impossible under neutrality are also impossible under selection. This condition needs to be respected when setting the initial conditions (ψ^U and ϕ^U at time zero). Over time, selection increases or decreases the probability of population states, genotypes or phenotypes that arise by reproduction with mutation, a by finite factor proportional to fitness. But selection cannot create entirely new states. On the other hand, some genotypes that arise by mutation can have zero fitness, and therefore be impossible under selection ($\psi^U(u) > 0$ but $\psi^U(u) = 0$).

When this happens, the corresponding term $\psi^U(u)\log_2\frac{\psi^U(u)}{\varphi^U(u)}$ is set to zero. Therefore this is a very natural assumption, and analogous arguments apply to joint/conditional distributions which we discuss next.

For a pair of variables U, V we can write their joint and conditional KL divergence (1),

$$D(U,V) = \sum_{u,v} \psi^{U,V}(u,v) \log_2 \frac{\psi^{U,V}(u,v)}{\varphi^{U,V}(u,v)}, \tag{S2}$$

$$D(U|V) = \sum_{v} \psi^{V}(v) \sum_{u} \psi^{U|V}(u|v) \log_2 \frac{\psi^{U|V}(u|v)}{\varphi^{U|V}(u|v)} \tag{S3}$$

where $\psi^{U,V}(u,v)$ and $\psi^{U|V}(u|v)$ are the joint and conditional probabilities under selection, and $\varphi^{U,V}(u,v)$ and $\varphi^{U|V}(u|v)$ under neutrality. With these definitions, the chain rule states two possible decompositions of the joint KL divergence,

$$D(U,V) = D(U) + D(V|U) = D(V) + D(U|V). \tag{S4}$$

S2. Population-level information: allele frequencies and LD

When there is a fixed number of loci, instead of genotype frequencies, an alternative way to describe a population state is in terms of allele frequencies. Allele frequencies by themselves, however, do not capture correlations between loci and therefore can miss some of the information that selection can accumulate on the population level. This can be expressed using the chain rule,

$$D(X) = D(\text{Allele freq.}) + D(X|\text{Allele freq.}), \tag{S5}$$

where the term $D(\text{Allele freq.}|X) = 0$ because regardless of selection, allele frequencies are fully determined by the genotype frequencies X. The term $D(X|\text{Allele freq.})$ quantifies how different from neutrality are the correlations between loci.

S3. Violation of the bound by Worden 1995 by drift

The genotype-level information introduced by Worden (2) (see Eq. (11) there) is the KL divergence between the genotype frequencies and a uniform distribution,

$$I = \sum_g x_g \log_2(M x_g), \tag{S6}$$

where M is the number of possible genotypes and x_g the frequency of genotype g (denoted q_j in (2)). Worden also introduced a similar genotype-level measure (Eq. (8) in (2), which was upper bounded by I. These measures of information can be seen as special cases of $D(G)$ and $D(Z)$ when there is no evolutionary stochasticity – ψ^X is concentrated at a single value x, and φ^X is concentrated at a single value of x that is uniform over all possible genotypes.

Worden proposes a bound on the rate of increase of I starting from a uniform x, and the maximal rate is proportional to a quantity similar to the genetic load, i.e. roughly a factor N (population size) times more stringent than the bound presented here.

The proof relies on the assumption that the population is large and x_g evolve deterministically, but later, validity in finite populations is claimed (Sec. 2.6 in (2)). This is mistaken: in a realistic population, I can hardly be zero to start with, as there will be more possible genotypes than individuals and x_g cannot be uniform. Starting from near uniform x, random drift will tend to remove variability from the population and concentrate all genotypes around some random ancestral genotype, and I will increase even without selection. This can also be seen as a consequence of the convexity of KL divergence: random fluctuations in x will, on average, increase I. This highlights the need to consider stochasticity as well as population variation when quantifying the intuitive notion of genetic information.

Worden's stringent bound does hold if the genotype frequencies evolve deterministically and there is no recombination. This is consistent with our observation that selection accumulates information less cost-efficiently when $Ns \gg 1$ and the fixation probability of a beneficial mutation is close to 1 (Main Text Fig. 3C,D).

S4. The single locus, two allele system used for figures

We use a haploid single locus, two allele system to produce Figures 1-5. The figures are produced with a Wright-Fisher model, Moran model (only the fitness flux in Fig. 4, S2B and S3), and some intuition can be gained by approximating it as diffusion under weak selection. Note that this is only an illustration, more general classes of models are discussed in sections S5, S6 and S7.

The system has two alleles, a and A, where the latter is beneficial under selection. It is parametrized by the population size N, mutation rate μ and selection coefficient $s(x)$ (which is frequency dependent only in Fig. 3C,D and S4).

S4A. Wright-Fisher model. Under the Wright-Fisher model, the state space is a set of discrete frequencies of the A allele, $x_A = 0,\ 1/N,\ 2/N,\ \dots,\ 1$, while the a allele always has the complementary frequency $x_a = 1 - x_A$. The two alleles have the following properties.

Allele g	frequency x_g	fitness $w_g(x)$	relative fitness $\hat{w}_g(x)$
a	$x_a = 1 - x_A$	$w_a(x) = 1$	$\hat{w}_a(x) = \frac{1}{1+s\,x_A}$
A	x_A	$w_a(x) = 1 + s$	$\hat{w}_A(x) = \frac{1+s}{1+s\,x_A}$

The probability of sampling allele A as a parent is $x_A \hat{w}_A(x)$, and the probability of sampling it as offspring is

$$q_A(x) = x_A(1-\mu) + x_a\mu \qquad [S7]$$

$$p_A(x) = x_A\hat{w}_A(x)(1-\mu) + x_a\hat{w}_a(x)\mu = \frac{x_A(1+s)(1-\mu) + (1-x_A)\mu}{1+sx_A}, \qquad [S8]$$

under neutrality and under selection respectively. The Wright-Fisher transition probabilities are given by the binomial distribution,

$$Q(x^{t+1}|x^t) = \binom{N}{Nx^{t+1}} q_A(x)^{Nx_A^{t+1}} (1-q_A(x))^{N-Nx_A^{t+1}}. \qquad [S9]$$

$$P(x^{t+1}|x^t) = \binom{N}{Nx^{t+1}} p_A(x)^{Nx_A^{t+1}} (1-p_A(x))^{N-Nx_A^{t+1}}. \qquad [S10]$$

This is a case of the Wright-Fisher model with selection among parents (see Eq. (S27,S28) and SI Sec. S5A.2). An analogous discrete-time Moran model can be written by plugging Eq. (S7,S8) into Eq. (S43,S44).

All calculations were done with $N \leq 200$. Given the small size of the system, we can compute the full matrix $P(x^{t+1}|x^t)$, and calculate the distribution over genotype frequencies over time by iterating $\psi^{X^{t+1}}(x^{t+1}) = \sum_{x^t} \psi^{X^t}(x^t)P(x^{t+1}|x^t)$.

S4B. The diffusion approximation. We write the diffusion approximation for the evolution of the frequency x_A. Following the notation in SI Sec. S7, the first two moments of change of x_A are given by

$$a_A = \mu(1-2\,x_A) \qquad \text{expected change due to mutation,} \qquad [S11]$$

$$a_A^s = \frac{s\,x_A(1-x_A)}{1+sx_A} \qquad \text{expected change due to selection,} \qquad [S12]$$

$$b_{AA} = \frac{x_A(1-x_A)}{N} \qquad \text{drift covariance.} \qquad [S13]$$

The diffusion equation for this system is a special case of Eq. (S57,S58).

Maintenance of information under weak selection. Since the diffusion process takes place along only one dimension, the stationary distribution can be determined by equating the probability flux to zero. For simplicity, we neglect the mean fitness $1 + sx_A \approx 1$ in the denominator in Eq. (S12), by assuming that selection is weak, $s \ll 1$.

The stationary distributions under selection and under neutrality are

$$\tilde{\psi} = \frac{(x_A(1-x_A))^{2N\mu-1}e^{2Nsx_A}}{Z(N,\mu,s)} \qquad \tilde{\varphi} = \frac{(x_A(1-x_A))^{2N\mu-1}}{Z(N,\mu,0)} \qquad [S14]$$

with the normalization constant

$$Z(N,\mu,s) = \int_0^1 (x_A(1-x_A))^{2N\mu-1}e^{2Nsx_A}dx_A = \Gamma(2N\mu)^2\,{}_1\tilde{F}_1(2N\mu;\,4N\mu;\,2Ns), \qquad [S15]$$

where Γ is the Gamma function and ${}_1\tilde{F}_1$ is the regularized confluent hypergeometric function.

Similar integrals yield results for the maintained information $D(X)$, $D(G)$ and the associated expected cost at the stationary state. We calculate the expectation of the cost $C(x) = \frac{V(x)}{2\ln 2} = \frac{s^2 x_A(1-x_A)}{2\ln 2}$ (see SI Sec. S9 and S7A). For the genotype-level information $D(G)$, we also need the expected frequency of A which is equal to its marginal probability, $\langle x_A \rangle = \tilde{\psi}^G(A)$. For brevity, we only write the leading terms in s for each quantity.

$$\langle C \rangle = \int_0^1 \tilde{\psi}\,C(x)\,dx_A = \frac{N\mu\,s^2}{(4N\mu+1)\,2\ln 2} + O\left(s^4\right) \qquad [S16]$$

$$\langle x_A \rangle = \tilde{\psi}^G(A) = \int_0^1 \tilde{\psi}\,x_A\,dx_A = \frac{1}{2} + \frac{Ns}{8N\mu+2} + O\left(s^3\right) \qquad [S17]$$

$$D(G) = \langle x_A \rangle \log_2\frac{\langle x_A \rangle}{1/2} + (1-\langle x_A \rangle)\log_2\frac{1-\langle x_A \rangle}{1/2} = \frac{(Ns)^2}{(4N\mu+1)^2\,2\ln 2} + O\left(s^4\right) \qquad [S18]$$

$$D(X) = \int_0^1 \tilde{\psi}\log_2\frac{\tilde{\psi}}{\tilde{\varphi}}\,dx_A = \frac{(Ns)^2}{(4N\mu+1)\,2\ln 2} + O\left(s^4\right) \qquad [S19]$$

Notably, at weak selection, both the cost $\langle C\rangle$ and the information $D(X)$, $D(G)$ scale with s^2. Their ratio is therefore given by the population size and the mutation rate,

$$\frac{D(G)}{\langle C\rangle} = \frac{N}{\mu\,(4N\mu+1)} + O(s^2), \qquad \text{[S20]}$$

$$\frac{D(X)}{\langle C\rangle} = \frac{N}{\mu} + O(s^2). \qquad \text{[S21]}$$

The ratio $\frac{N}{\mu\,(4N\mu+1)}$ is shown in Fig. 5C.

S5. The bound on information accumulation rate – Markov chains

The bound on information accumulation rate, as stated in Main Text Eq. (10,11) holds across several different model classes. Here we derive it for models that are Markov chain, in particular the Wright-Fisher model and the discrete Moran model. The two following sections contain similar derivations for continuous time Markov chains and the diffusion approximation. Note that all of the model parameters, such as those that describe selection, mutation or population size, can be time dependent, but we do not write it explicitly as we only need to focus on a single time step.

In the Markov chains class of models, the population state X^t takes discrete values x^t at discrete time steps t. The distribution over states is governed by

$$\varphi^{X^{t+1}}(x^{t+1}) = \sum_{x^t} Q(x^{t+1}|x^t)\,\varphi^{X^t}(x^t) \qquad \text{under neutrality,} \qquad \text{[S22]}$$

$$\psi^{X^{t+1}}(x^{t+1}) = \sum_{x^t} P(x^{t+1}|x^t)\,\psi^{X^t}(x^t) \qquad \text{under selection,} \qquad \text{[S23]}$$

where $\varphi^{X^t}(x^t)$ and $\psi^{X^t}(x^t)$ are the marginal distributions over population states at time t, and $Q(x^{t+1}|x^t) = \varphi^{X^{t+1}|X^t}(x^{t+1}|x^t)$ and $P(x^{t+1}|x^t) = \psi^{X^{t+1}|X^t}(x^{t+1}|x^t)$ are the transition probabilities. $Q(x^{t+1}|x^t)$ and $P(x^{t+1}|x^t)$, as well as all the parameters that we later introduce to specify them, can be time-dependent, but we do not write it explicitly. The population-level information at time t is

$$D(X^t) = \sum_{x^t} \psi^{X^t}(x^t)\log_2\frac{\psi^{X^t}(x^t)}{\varphi^{X^t}(x^t)}. \qquad \text{[S24]}$$

In general, the chain rule Eq. (S4) yields a bound

$$\Delta D(X^t) = D(X^{t+1}) - D(X^t) = D(X^{t+1}|X^t) - D(X^t|X^{t+1}) \qquad \text{[S25]}$$

$$\le D(X^{t+1}|X^t) = \sum_{x^t}\psi^{X^t}(x^t)\sum_{x^{t+1}} P(x^{t+1}|x^t)\log_2\frac{P(x^{t+1}|x^t)}{Q(x^{t+1}|x^t)}. \qquad \text{[S26]}$$

The expression $\le D(X^{t+1}|X^t)$ corresponds to the expected KL cost of control (3, 4). In the special case when $Q(x^{t+1}|x^t)$ and $P(x^{t+1}|x^t)$ are independent of time $\psi^{X^t}(x^t)$ is the stationary distribution of $P(x^{t+1}|x^t)$, $D(X^{t+1}|X^t)$ is also the KL divergence rate between $P(x^{t+1}|x^t)$ and $Q(x^{t+1}|x^t)$. We now examine specific forms of the transition probabilities given by the Wright-Fisher model and the Moran model.

S5A. Wright-Fisher model. In this general model, each time step t represents a generation, and consists of sampling a new population of N offspring genotypes that constitute the population at $t+1$. The basic assumption is that the offspring genotypes are sampled independently with with probabilities $q_g(x^t)$ without selection or $p_g(x^t)$ with selection, leading to multinomial probability distributions over the frequencies x^{t+1} in the next generation,

$$Q(x^{t+1}|x^t) = \binom{N}{Nx^{t+1}}\prod_g q_g(x^t)^{Nx_g^{t+1}}, \qquad \text{[S27]}$$

$$P(x^{t+1}|x^t) = \binom{N}{Nx^{t+1}}\prod_g p_g(x^t)^{Nx_g^{t+1}}, \qquad \text{[S28]}$$

where $\binom{N}{Nx^{t+1}} = \frac{N!}{\prod_g (Nx_g^{t+1})!}$ is the multinomial coefficient. We can use these expressions to write down the general bound Eq. (S26) as

$$\Delta D(X^t) \le D(X^{t+1|X^t}) = N\sum_{x^t}\psi^{X^t}(x^t)\sum_g p_g(x^t)\log_2\frac{p_g(x^t)}{q_g(x^t)} \qquad \text{[S29]}$$

The probabilities $q_g(x^t)$ capture arbitrary mutation and recombination, and we show examples of the form $q_g(x^t)$ can take below. Selection is modelled by the relationship between $q_g(x^t)$ and $p_g(x^t)$, and this can be done in two ways, which we discuss in the following subsections.

Asexual reproduction. In an asexual population with mutation, $q_g(x)$ will have the form

$$q_g(x) = \sum_{g'} x_{g'}\, \rho^{\text{asex}}_{g'g}, \tag{S30}$$

where $\rho^{\text{asex}}_{g'g}$ is the probability that a parent with genotype g' produces offspring with genotype g, and includes arbitrary mutation.

Sexual reproduction, random mating. Provided that recombination happens always between two parental genotypes, we sum over possible pairs of parental genotypes,

$$q_g(x) = \sum_{g_1 g_2} x_{g_1} x_{g_2}\, \rho^{\text{rec}}_{g_1 g_2 g} \tag{S31}$$

with $x_{g_1} x_{g_2}$ being the probability of a parental pair g_1, g_2 and $\rho^{\text{rec}}_{g_1 g_2 g}$ the probability that this pair produces offspring with genotype g. This includes arbitrary mutation and recombination.

Alternatively, we can distinguish between two sexes, classifying each genotype as male ($g \in \mathcal{G}^M$) of female ($g \in \mathcal{G}^F$). We sum over all male-female pairs,

$$q_g(x) = \sum_{g_m \in \mathcal{G}^M} \sum_{g_f \in \mathcal{G}^F} \frac{x_{g_m} x_{g_f}}{Z(x)}\, \rho^{\text{sex}}_{g_m g_f g} \tag{S32}$$

with $\rho^{\text{sex}}_{g_m g_f g}$ being the probability that parents g_m, g_f give rise to offspring g and

$$Z(x) = \sum_{g_m \in \mathcal{G}^M} \sum_{g_f \in \mathcal{G}^F} x_{g_m} x_{g_f} \tag{S33}$$

is a normalization factor such that $x_{g_m} x_{g_f}/Z(x)$ is the probability of a parental pair g_m, g_f.

Nonrandom mating. In the case of sexual reproduction, we can replace the expression $x_{g_m} x_{g_f}/Z(x)$ by a different expression for the probability of sampling a mating pair g_m, g_f. For example, we can include a factor $0 \le \sigma_{g_m g_f} \le 1$ corresponding to the probability that individuals with this pair of genotypes will mate. Then $q_g(x)$ will have the form

$$q_g(x) = \sum_{g_m \in \mathcal{G}^M} \sum_{g_f \in \mathcal{G}^F} \frac{x_{g_m} x_{g_f} \sigma_{g_m g_f}}{\tilde{Z}(x)}\, \rho^{\text{sex}}_{g_m g_f g}, \tag{S34}$$

with normalization

$$\tilde{Z}(x) = \sum_{g_m \in \mathcal{G}^M} \sum_{g_f \in \mathcal{G}^F} x_{g_m} x_{g_f} \sigma_{g_m g_f}. \tag{S35}$$

S5A.1. Selection in an infinite offspring pool. Here we assume that all individuals (under asexual reproduction) or all pairs of individuals (under sexual reproduction) in the population at time t contribute a large number of offspring to a common pool. The next generation then consists of N individuals sampled from this pool, with or without selection.

The genotype frequencies in the pool will be equal to $q_g(x^t)$, since this is the probability that a random invididual (or pair of individuals) from the population x^t has offspring with genotype g. Under neutrality, the genotypes that survive and constitute the next generation are sampled at random. Under selection, genotypes from the pool are sampled with probabilities proportional to fitness $w_g(x^t)$, leading to

$$p_g(x^t) = \frac{q_g(x^t) w_g(x^t)}{\sum_{g'} q_{g'}(x^t)\, w_{g'}(x^t)} = q_g(x^t)\, \tilde{w}_g(x^t). \tag{S36}$$

where $\tilde{w}_g(x^t) = \frac{w_g(x^t)}{\sum_{g'} q_{g'}(x^t)\, w_{g'}(x)}$ is the relative fitness of g calculated within the offspring pool where selection takes place. Combining it with Eq. (S29), we obtain the bound

$$\Delta D(X^t) \le D(X^{t+1}|X^t) = N \sum_{x^t} \psi^{X^t}(x^t) \sum_g q_g(x^t)\, \tilde{w}_g(x^t) \log_2 \tilde{w}_g(x^t) = N \langle C^t_{\text{pool}} \rangle, \tag{S37}$$

where the last expression coincides with the definition of the cost of selection in Main Text Eq. (S89), calculated at time t within the offspring pool where selection takes place, and averaged over possible population states x^t.

S5A.2. Selection among parents. Here we assume that selection takes place before reproduction. From the population at time t, we first sample N genotypes (under asexual reproduction) or N pairs of genotypes (under sexual reproduction) as parents. These are sampled independently, with probabilities proportional to fitness. Then we sample one offspring genotype for each parent/pair of parents, with mutation and recombination, and these constitute the population at time $t+1$.

When sampling genotypes as parents, g gets picked with probability given by its frequency x_g^t under neutrality, and $x_g^t \hat{w}_g(x^t)$ under selection where $\hat{w}_g(x^t) = \frac{w_g(x^t)}{\sum_{g'} x_{g'} w_{g'}(x)}$ is the relative fitness of genotype g, now computed within the adult population at time t.

We can rewrite Eq. (S29) with

$$p_g(x^t) = q_g\left(\hat{w}(x^t) \circ x^t\right), \qquad \text{[S38]}$$

where $(\hat{w}(x^t) \circ x^t)_g = \hat{w}_g(x^t)\, x_g^t$ is the vector of genotype frequencies that are weighted by their relative fitness. However, Eq. (S38) is difficult to analyse. An easier approach is to introduce an intermediate variable Y^t, which signifies the genotype frequencies among parents (or pairs of parents) at generation t. The Wright-Fisher model is a Markov chain of the form

$$\cdots \to X^t \to Y^t \to X^{t+1} \to Y^{t+1} \to \cdots. \qquad \text{[S39]}$$

Selection operates in the step $X^t \to Y^t$ when parents are sampled from the existing population, but not in the step $Y^t \to X^{t+1}$ where reproduction takes place with mutation and recombination. This can be expressed by writing the chain rule

$$D(X^{t+1}|X^t) = D(Y^t|X^t) + D(X^{t+1}|Y^t, X^t) - D(Y^t|X^{t+1}, X^t) \leq D(Y^t|X^t) \qquad \text{[S40]}$$

where the term $D(X^{t+1}|Y^t, X^t) = D(X^{t+1}|Y^t) = 0$ because $Y^t \to X^{t+1}$ is the reproduction step with no selection. The term $D(Y^t|X^{t+1}, X^t)$ is nonnegative and can be dropped since an upper bound on $D(X^{t+1}|X^t)$ is sufficient for our purposes, but deserves some attention as it could make the bound loose. $D(Y^t|X^{t+1}, X^t)$ can only be large when, given the genotype frequencies X^t, X^{t+1} at two subsequent generations, there is uncertainty about the genotype frequencies among the parents Y^t sampled from X^t that gave rise to X^{t+1}. In diverse populations with low mutation rates or large genomes, parents tend to be easy to identify, and $D(Y^t|X^{t+1}, X^t)$ will be small.

Finally, to calculate $D(Y^t|X^t)$ we note that conditionally on X^t, Y^t is a multinomial variable with kN trials ($k=1$ under asexual reproduction or $k=2$ under sexual reproduction) and probabilities x_g^t under neutrality or $x_g^t \hat{w}_g(x^t)$ under selection. Then the bound can be written as

$$\Delta D(X^t) \leq D(Y^t|X^t) = kN \sum_{x^t} \psi^{X^t}(x^t) \sum_g x_g^t\, \hat{w}_g(x^t) \log_2 \hat{w}_g(x^t) = kN\langle C^t\rangle. \qquad \text{[S41]}$$

Here we have assumed no distinction between sexes, but we can extend to that case by sampling N parents of each sex separately and find

$$\Delta D(X^t) \leq D(Y^t|X^t) = N\langle C^t_{\text{male}}\rangle + N\langle C^t_{\text{female}}\rangle. \qquad \text{[S42]}$$

S5B. Discrete-time Moran model. This model can be defined similarly to the Wright-Fisher model, but under the Moran model each time step consists of only one birth and one death, and there are N such time steps per generation.

The genotype that dies is chosen at random from the population x^t, and the probability that it will be g is equal to its frequency x_g^t. The probability that the genotype born is g' is $q_{g'}(x^t)$ under neutrality and $p_{g'}(x^t)$ under selection. These can take the same form as under the Wright-Fisher model, with selection within an infinite offspring pool or among parents. This gives rise to the transition probabilities

$$Q(x^{t+1}|x^t) = \begin{cases} x_g^t q_{g'}(x^t); & x^{t+1} = x^t + \frac{e^{g'}}{N} - \frac{e^g}{N},\ g' \neq g \quad (g \text{ dies, } g' \text{ born}), \\ \sum_g x_g^t q_g(x^t); & x^{t+1} = x^t, \\ 0; & \text{otherwise}, \end{cases} \qquad \text{[S43]}$$

$$P(x^{t+1}|x^t) = \begin{cases} x_g^t p_{g'}(x^t); & x^{t+1} = x^t + \frac{e^{g'}}{N} - \frac{e^g}{N},\ g' \neq g \quad (g \text{ dies, } g' \text{ born}), \\ \sum_g x_g^t p_g(x^t); & x^{t+1} = x^t, \\ 0; & \text{otherwise}, \end{cases} \qquad \text{[S44]}$$

where e^g is a vector of genotype frequencies with one at element g and zeros elsewhere. Now the bound on information accumulation rate, Eq. (S26), simplifies to

$$\Delta D(X^t) \leq D(X^{t+1}|X^t) = \sum_{x^t} \psi^{X^t}(x^t) \left(\sum_g p_g(x^t) \log_2 \frac{p_g(x^t)}{q_g(x^t)} - \sum_g \beta_g(x^t) \log_2 \frac{\beta_g(x^t)}{\alpha_g(x^t)} \right) \qquad \text{[S45]}$$

$$\leq \sum_{x^t} \psi^{X^t}(x^t) \sum_g p_g(x^t) \log_2 \frac{p_g(x^t)}{q_g(x^t)}, \qquad \text{[S46]}$$

$$\leq kN\langle C^t\rangle \qquad \text{[S47]}$$

where we have used $\alpha_g(x^t) = \frac{x_g^t q_g(x^t)}{\sum_{g'} x_{g'}^t q_{g'}(x^t)}$ and $\beta_g(x^t) = \frac{x_g^t p_g(x^t)}{\sum_{g'} x_{g'}^t p_{g'}(x^t)}$ to denote the probability that if the genotype that is born and dies is the same, it is the genotype g. Even though selection makes $\beta_g(x^t)$ different from $\alpha_g(x^t)$, such replacements leave the population unchanged regardless of g, and this is associated with the nonpositive second term inside the brackets in Eq. (S45), which reduces the amount of information that can be accumulated.

Eq. (S46) is almost identical to the bound on information accumulation rate under the Wright-Fisher model, Eq. (S29). This means that the discussion of the two models of selection in Sec. S5A.1 and S5A.2 also applies to the Moran model, which allows us to write Eq. (S47). The only difference is that in each time step, we sample only one genotype from the offspring pool, or one parent (or pair of parents). This is reflected in the missing factor N. As there are N time steps per generation, the bound on information accumulated per generation again scales with $kN\langle C^t\rangle$.

Note, however, that the effective population size of the Moran model (i.e. the population size of a Wright-Fisher model with the same covariance in allele frequency change per generation) is $N_e = N/2$. This is because in the Moran model, both the births and deaths are random events, whereas the Wright-Fisher model only has random births. We can therefore expect the tighter bound $\Delta D(X^t) \lesssim kN_e\langle C^t\rangle$ to hold for large enough populations, as both models approach the same diffusion limit. We also prove the bound under the diffusion approximation separately in Sec. S7.

S6. The bound on information accumulation rate – continuous-time Markov chains

In this class of models, the genotype frequencies X^t are discrete, but the time t is continuous. The distributions $\varphi^{X^t}(x)$, $\psi^{X^t}(x)$ over X^t are governed by the master equations

$$\frac{d}{dt}\varphi^{X^t}(x) = \sum_{x'} \bar{Q}(x,x')\,\varphi^{X^t}(x') \qquad \text{under neutrality,} \tag{S48}$$

$$\frac{d}{dt}\psi^{X^t}(x) = \sum_{x'} \bar{P}(x,x')\,\psi^{X^t}(x') \qquad \text{under selection,} \tag{S49}$$

where $\bar{Q}(x,x')$ and $\bar{P}(x,x')$ are transition rates from x' to x under neutrality and selection respectively. These can be time dependent. The population-level information $D(X^t)$ is defined as in Eq. (S24), but now changes continuously in time. The rate of this change is upper bounded as

$$\frac{d}{dt}D(X^t) \leq \sum_x \psi^{X^t}(x) \sum_{x,\,x'\neq x} \left(\bar{P}(x',x)\log\frac{\bar{P}(x',x)}{\bar{Q}(x',x)} + \bar{Q}(x',x) - \bar{P}(x',x)\right). \tag{S50}$$

When $\bar{P}$ and $\bar{Q}$ are independent of time and $\psi^{X^t}(x)$ is the stationary distribution associated with $\bar{P}$, the right hand side corresponds to the KL divergence rate between $\bar{P}$ and $\bar{Q}$, as derived in (5). The bound Eq. (S50) can be verified algebraically, or derived from the discrete bound Eq. (S26) by taking $Q = e^{\epsilon\bar{Q}}$, $P = e^{\epsilon\bar{P}}$ and the limit $\epsilon \to 0$. We now show an example of the form that $\bar{P}$ and $\bar{Q}$ can take.

S6A. Continuous-time Moran model. This model is based on its discrete-time counterpart in Sec. S5B. Only transitions consisting of replacing one genotype (g, death) by another (g', birth) are allowed, and the transition rates have the form

$$\bar{Q}(x',x) = \begin{cases} Nx_g q_{g'}(x); & x' = x - \frac{e^g}{N} + \frac{e^{g'}}{N},\ g' \neq g, \\ -\sum_{g,g'\neq g} Nx_g q_{g'}(x); & x' = x, \\ 0; & \text{otherwise,} \end{cases} \tag{S51}$$

$$\bar{P}(x',x) = \begin{cases} Nx_g p_{g'}(x); & x' = x - \frac{e^g}{N} + \frac{e^{g'}}{N},\ g' \neq g, \\ -\sum_{g,g'\neq g} Nx_g p_{g'}(x); & x' = x, \\ 0; & \text{otherwise,} \end{cases} \tag{S52}$$

where time is measured in generations, i.e. there are on average N replacement events per unit time. With this form of the transition rates, we can rewrite the general bound Eq. (S50) as

$$\frac{d}{dt}D(X^t) \leq N\sum_x \psi^{X^t}(x) \sum_{g,g'\neq g} x_g\left(p_{g'}(x)\log\frac{p_{g'}(x)}{q_{g'}(x)} + q_{g'}(x) - p_{g'}(x)\right) \tag{S53}$$

$$= N\sum_x \psi^{X^t}(x)\sum_g\left(p_g(x)\log\frac{p_g(x)}{q_g(x)} - x_g q_g(x)\left(\frac{p_g(x)}{q_g(x)}\log\frac{p_g(x)}{q_g(x)} + 1 - \frac{p_g(x)}{q_g(x)}\right)\right) \tag{S54}$$

$$\leq N\sum_x \psi^{X^t}(x)\sum_g p_g(x)\log\frac{p_g(x)}{q_g(x)}. \tag{S55}$$

This is the same bound as for the discrete Moran model in Eq. (S47), up to the factor N, which is due to the different unit of time.

S7. The bound on information accumulation rate – diffusion approximation

Here we show an upper bound on the rate of accumulation of information under the diffusion approximation. The approach is similar to Iwasa (6) (who assumed detailed balance) and Hasegawa (7) (who did not), but here we distinguish between the processes with and without selection. We start by deriving a general bound for a pair of diffusion processes, and then apply it to the population genetics context in Sec. S7A.

For brevity, we write the probability density over population states x at time t as $\varphi = \varphi(x,t)$ under neutrality and $\psi = \psi(x,t)$ under selection. The population-level information is now determined by integration,

$$D(X) = \int \psi \log_2 \frac{\psi}{\varphi}\, dx = \frac{1}{\ln 2} \int \psi \ln \frac{\psi}{\varphi}\, dx. \qquad \text{[S56]}$$

Note that while we stick to measuring information in bits, it is more convenient to use the natural logarithm during the derivation.

The diffusion equation is parametrized by the first and second moment of change in x_g. Selection is assumed to only exert control through the first moment, which we label as a_g under neutrality and $a_g + a_g^s$ under selection. The second moment is $b_{gg'}$, both under neutrality and under selection. All these are functions of x and t, e.g. $a_g = a_g(x,t)$, but we do not write this for brevity. We sum over any index that appears twice in a term, e.g. $\partial_g b_{g'g} = \sum_{g=1}^{\mathcal{G}-1} \partial_g b_{g'g}$. Note that the diffusion is described in the subspace of $\mathcal{G}-1$ genotype frequencies, where $\mathcal{G}$ is the number of genotypes – the last frequency is determined by normalization, $x_{\mathcal{G}} = 1 - \sum_{g=1}^{\mathcal{G}-1} x_g$. The diffusion equation is

$$\partial_t \varphi = -\partial_g \left(u_g \varphi\right), \qquad u_g = a_g \qquad - \frac{1}{2}\partial_{g'} b_{gg'} - \frac{1}{2} b_{gg'} \partial_{g'} \ln \varphi \qquad \text{under neutrality,} \qquad \text{[S57]}$$

$$\partial_t \psi = -\partial_g \left(v_g \psi\right), \qquad v_g = a_g + a_g^s - \frac{1}{2}\partial_{g'} b_{gg'} - \frac{1}{2} b_{gg'} \partial_{g'} \ln \psi \qquad \text{under selection,} \qquad \text{[S58]}$$

where we introduced the velocity fields $u_g = u_g(x,t)$ and $v_g = v_g(x,t)$ such that $u_g\varphi$ and $v_g\psi$ are the probability fluxes under neutrality and under selection respectively. From their definition, it follows that

$$\partial_g \ln \frac{\psi}{\varphi} = -2 b_{gg'}^{-1} \left(v_{g'} - u_{g'} - a_{g'}^s\right). \qquad \text{[S59]}$$

The rate of change of $D(X)$ can be written as

$$\frac{d}{dt} D(X) = \frac{1}{\ln 2} \int \partial_t \left(\psi \ln \frac{\psi}{\varphi}\right) dx, \qquad \text{[S60]}$$

and the integrand can be written as

$$\partial_t \left(\psi \ln \frac{\psi}{\varphi}\right) = \ln \frac{\psi}{\varphi}\, \partial_t \psi + \partial_t \psi - \frac{\psi}{\varphi}\, \partial_t \varphi \qquad \text{[S61]}$$

$$= -\ln \frac{\psi}{\varphi}\, \partial_g \left(v_g \psi\right) - \partial_g \left(v_g \psi\right) + \frac{\psi}{\varphi}\, \partial_g \left(u_g \varphi\right) \qquad \text{[S62]}$$

$$= -\partial_g \left(v_g \psi \ln \frac{\psi}{\varphi} + \left(v_g - u_g\right) \psi\right) + \psi \left(v_g - u_g\right) \partial_g \ln \frac{\psi}{\varphi}. \qquad \text{[S63]}$$

The first term is a divergence and vanishes after integration in Eq. (S60) because $u_g\varphi$ and $v_g\psi$ cannot cross the domain boundary (assuming $\psi/\varphi < \infty$). Therefore

$$\frac{d}{dt} D(X) = \frac{1}{\ln 2} \int \psi \left(v_g - u_g\right) \partial_g \ln \frac{\psi}{\varphi}\, dx \qquad \text{[S64]}$$

$$= -\frac{2}{\ln 2} \int \psi \left(v_g - u_g\right) b_{gg'}^{-1} \left(v_{g'} - u_{g'} - a_{g'}^s\right) dx \qquad \text{[S65]}$$

$$= -\frac{2}{\ln 2} \int \psi \left(v_g - u_g - \frac{1}{2} a_g^s\right) b_{gg'}^{-1} \left(v_{g'} - u_{g'} - \frac{1}{2} a_{g'}^s\right) dx + \frac{1}{2 \ln 2} \int \psi\, a_g^s\, b_{gg'}^{-1}\, a_{g'}^s\, dx. \qquad \text{[S66]}$$

The last expression has two terms, both of which are quadratic forms. The first one makes a nonpositive contribution, leading to the upper bound in information accumulation rate,

$$\frac{d}{dt} D(X) \leq \frac{1}{2 \ln 2} \int \psi\, a_g^s\, b_{gg'}^{-1}\, a_{g'}^s\, dx. \qquad \text{[S67]}$$

On the right hand side we can identify the KL cost of control (4) in bits. This bound holds for any pair of diffusion processes with the same fluctuations covariance $b_{gg'}$. We will now discuss it in the context of population genetics.

S7A. Application to population genetics. The bound Eq. (S67) does not depend on the form of a_g, and therefore it can be used to model arbitrary mutation and recombination, for example by taking

$$a_g = q_g - x_g \qquad \text{[S68]}$$

with $q_g = q_g(x)$ as introduced in the discrete models above. Notably, a_g does not need to be the gradient of a scalar potential.

The fluctuations in genotype frequencies can modeled based on multinomial sampling in the Wright-Fisher model (Eq. (S27,S28)),

$$b_{gg'} = \frac{\delta_{gg'} x_g - x_g x_{g'}}{N}, \qquad \text{[S69]}$$

with no summation over g, and where the (effective) population size N can be time-dependent. This has the inverse (8)

$$b^{-1}_{gg'} = N\left(\frac{\delta_{gg'}}{x_g} + \frac{1}{x_{\mathcal{G}}}\right). \qquad \text{[S70]}$$

The control term a^s_g imposed by selection can be written as

$$a^s_g = (\hat{w}_g - 1)\, x_g \qquad \text{where} \qquad \hat{w}_g = \frac{w_g}{\sum_g w_g x_g} \qquad \text{[S71]}$$

where w_g is the fitness of genotype g, possibly time and frequency dependent, and $\hat{w}_g$ is the relative fitness. With these definitions, we find that

$$\sum_{g,g'=1}^{\mathcal{G}-1} a^s_g\, b^{-1}_{gg'}\, a^s_{g'} = N \sum_{g=1}^{\mathcal{G}} \frac{(a^s_g)^2}{x_g} = N \sum_{g=1}^{\mathcal{G}} (\hat{w}_g - 1)^2\, x_g = NV(x) \qquad \text{[S72]}$$

and the bound on information accumulation rate is

$$\frac{d}{dt} D(X) \leq \frac{N\langle V\rangle}{2\ln 2} = N\langle C\rangle. \qquad \text{[S73]}$$

In the last equation, we equate $\frac{\langle V\rangle}{2\ln 2} = \langle C\rangle$ – we show that this holds under weak selection in Sec. S9.

We can get intuition about the tightness of this bound by analyzing the first, nonpositive term in Eq. (S66). The bound is only tight when $v_g - u_g - \frac{1}{2}a^s_g = 0$ for all x with nonzero ψ. An interesting specific case is when the neutral process is at an equilibrium with detailed balance, such that $u_g = 0$. We note that v_g can be decomposed as $v_g = v'_g + a^s_g$ into the contribution from selection a^s_g and all other evolutionary forces, v'_g. Our bound is then tight when $a^s_g = -2v'_g$, i.e. when selection induces a probability flux in exactly the opposite direction and exactly twice the magnitude as the all the other evolutionary forces combined. While this might occasionally and approximately be the case, it will only be a transient phenomenon.

Suppose that the population starts at the neutral equilibrium with $v'_g = 0$ and then selection starts to act, e.g. after a change in the environment. For any nonzero a^s_g, the bound cannot be tight as $a^s_g \neq -2v'_g = 0$. After some time a new equilibrium might be reached, with $v_g = v'_g + a^s_g = 0$, where again, the bound is not tight $a^s_g \neq -2v'_g = 2a^s_g$. In this case, maintenance costs are incurred but no further adaptation takes place. The bound can only be tight for a moment when adaptation is taking place, selection pulls the population in the opposite direction as the other evolutionary forces combined, but selection is twice as strong, $a^s_g = -2v'_g$.

S8. Relationship with free fitness and statistical physics

Stochastic models in population genetics show some mathematical properties analogous to statistical physics. In particular, a quantity called free fitness, analogous to free energy in physics, can be defined and shown to monotonically increase over time. In this section we provide some background about free fitness and discuss two connections with our work. First, the increasing property of free fitness can be proved by a method similar to the proof of our bound on information accumulation rate. Second, free fitness can be written as the difference between mean log fitness and genetic information as defined in this paper (e.g. on the genotype or population level), implying that evolution tends to maximize mean log fitness at a given amount of information.

S8A. Boltzmann form of stationary distributions. Under suitable conditions, the stationary distributions in population genetics models take a form similar to the Boltzmann distribution. Models on both the population level and the genotype level display this property.

- If mutation is weak ($NU \ll 1$ where U is the total mutation rate across the studied genomic region), populations are mostly monomorphic, with only occasional fixations of a different genotype. The system can then be described with the most recently fixed genotype g and the distribution $\psi^G(g)$. The stationary distribution $\tilde{\psi}^G(g)$ takes the form (9, 10)

$$\tilde{\psi}^G(g) = \frac{1}{Z^G} e^{2N \ln w_g}, \qquad \text{[S74]}$$

  where $2N$ is again analogous to inverse temperature, log fitness $\ln w_g$ is analogous to negative energy, and Z^G is normalization constant.

- Assuming many biallelic loci under linkage equilibrium, we can describe the system with the vector of allele frequencies p and the joint distribution $\psi^P(p)$ over them. The stationary distribution $\tilde{\psi}^P(p)$ can be derived from the diffusion approximation and takes the form (11, 12)

$$\tilde{\psi}^P(p) = \frac{1}{Z^P} \prod_i (p_i q_i)^{2N\mu - 1} e^{2N \ln \bar{w}(p)}, \qquad \text{[S75]}$$

where p_i and $q_i = 1 - p_i$ are the allele frequencies of the two alleles at locus i and Z^P is a normalization constant. Twice the population size $2N$ takes the role of inverse temperature and log mean fitness $\ln \bar{w}(p)$ takes the role of negative energy. The factors $(p_i(1-p_i))^{2N\mu-1}$ correspond to mutation and drift potential (similar to e.g. chemical potential), which will be made clearer in the next subsection.

The formulas apply to haploids, but similar formulas apply to diploids or when mutation coefficients vary across loci or alleles (see e.g. the SI of (10)). Importantly, they depend on the assumption of detailed balance. At stationarity, net probability flux between any two allele frequency vectors or genotypes must be zero, making $\tilde{\psi}^G(g)$ and $\tilde{\psi}^P(p)$ equilibrium distributions. This can be violated under certain forms of mutation, recombination and strong selection, leading to additional terms related to robustness (13).

S8B. Free fitness. When a system starts from an arbitrary initial distribution and approaches the stationary distribution in Eq. (S75) or Eq. (S74), we can track this progress using free fitness (6, 10), which increases monotonically in time as was shown previously and as we can also prove in more generality here.

- On the genotype level, we can define free fitness F^G at any distribution $\psi^G(g)$ away from equilibrium as a sum of expected log fitness and entropy terms,

$$F^G = \underbrace{\langle \ln w_g \rangle_{\psi^G}}_{\text{Selection}} + \underbrace{\frac{1}{2N} \left\langle \ln \frac{1}{\psi^G(g)} \right\rangle_{\psi^G}}_{\text{Entropy}} \qquad \text{[S76]}$$

$$= \underbrace{\frac{\ln Z^G}{2N}}_{\substack{\text{Equilibrium} \\ \text{free fitness}}} - \underbrace{\frac{1}{2N} \left\langle \ln \frac{\psi^G(g)}{\tilde{\psi}^G(g)} \right\rangle_{\psi^G}}_{\substack{\text{KL divergence} \\ \text{from equilibrium}}} = \tilde{F}^G - \frac{1}{2N} D_{KL}(\psi^G || \tilde{\psi}^G) \qquad \text{[S77]}$$

where $\langle \cdot \rangle_{\psi^G}$ denotes an expectation over $g \sim \psi^G(g)$. In the special case of equilibrium $\psi^G = \tilde{\psi}^G$, we obtain $F^G = \tilde{F}^G = \frac{\ln Z^G}{2N}$, and away from equilibrium, free fitness is reduced by an amount proportional to the KL divergence between the actual distribution ψ^G and the equilibrium $\tilde{\psi}^G$.

- On the population level with linkage equilibrium, free fitness at a distribution ψ^P can be defined as a sum of three terms – a negative potential for selection, mutation and drift, and entropy:

$$F^P = \underbrace{\langle \ln \bar{w}(p) \rangle_{\psi^P}}_{\text{Selection}} + \underbrace{\frac{2N\mu - 1}{2N} \left\langle \sum_i \ln(p_i q_i) \right\rangle_{\psi^P}}_{\text{Mutation and drift}} + \underbrace{\frac{1}{2N} \left\langle \ln \frac{1}{\psi^P(p)} \right\rangle_{\psi^P}}_{\text{Entropy}} \qquad \text{[S78]}$$

$$= \underbrace{\frac{\ln Z^P}{2N}}_{\substack{\text{Equilibrium} \\ \text{free fitness}}} - \underbrace{\frac{1}{2N} \left\langle \ln \frac{\psi^P(p)}{\tilde{\psi}^P(p)} \right\rangle_{\psi^P}}_{\substack{\text{KL divergence} \\ \text{from equilibrium}}} = \tilde{F}^P - \frac{1}{2N} D_{KL}(\psi^P || \tilde{\psi}^P) \qquad \text{[S79]}$$

where the expectations $\langle \cdot \rangle$ are taken over $p \sim \psi^P(p)$. While mutation and drift now appear as additional terms in free fitness, free fitness can again be decomposed into its value at equilibrium and a difference proportional to the KL divergence away from it.

The key property of the free fitness is that it is a non-decreasing function of time, until it is maximized at equilibrium. This was proved by Iwasa (6) for the case of F^P and Sella and Hirsh (10) for the case of F^G. In the next section we show that both results can also be derived by the same method as our bound on information accumulation rate.

S8C. Monotonic convergence of stochastic processes to their stationary distributions. The general bounds on information accumulation rate (Eq. (S26) for Markov chains, Eq. (S50) for continuous time Markov chains and Eq. (S67) for the diffusion approximation) apply for any pair of stochastic processes, provided that they have compatible support such that the KL divergence is well defined. To make this explicit, we focus on the case of discrete Markov chains, consider some general process ξ instead of the neutral process φ, and introduce notation that generalizes $D(X^t)$,

$$D_{\psi||\xi}(X^t) = \sum_{x^t} \psi^{X^t}(x^t) \log_2 \frac{\psi^{X^t}(x^t)}{\xi^{X^t}(x^t)} \quad \text{[S80]}$$

$$D_{\psi||\xi}(X^{t+1}|X^t) = \sum_{x^t} \psi^{X^t}(x^t) \sum_{x^{t+1}} \psi^{X^{t+1}|X^t}(x^{t+1}|x^t) \log_2 \frac{\psi^{X^{t+1}|X^t}(x^{t+1}|x^t)}{\xi^{X^{t+1}|X^t}(x^{t+1}|x^t)}. \quad \text{[S81]}$$

The KL divergence chain rule now yields the inequality

$$\Delta D_{\psi||\xi}(X^t) = D_{\psi||\xi}(X^{t+1}) - D_{\psi||\xi}(X^t) \leq D_{\psi||\xi}(X^{t+1}|X^t). \quad \text{[S82]}$$

If $\xi = \varphi$ is the neutral process, this is the KL cost of selection bound on the information accumulation rate (Eq. (S26) and Main Text Eq. (9). But we can also choose ξ such that it does contain selection and has the same transition probabilities as ψ, but starts from a different initial condition, i.e. $\xi^{X^{t+1}|X^t} = \psi^{X^{t+1}|X^t}$ and $\xi^{X^0} \neq \psi^{X^0}$. Then we find that $D_{\psi||\xi}(X^{t+1}|X^t) = 0$ and $\Delta D_{\psi||\xi}(X^t) \leq 0$, i.e. the divergence between ψ^{X^t} and ξ^{X^t} is non-increasing over time, because relative to ξ, there is no control exerted on ψ.

If, in addition, the system has a unique stationary distribution $\tilde{\psi}^X$ and ξ^{X^0} is initialized there (and therefore stays there indefinitely, $\xi^{X^0} = \xi^{X^t} = \tilde{\psi}^X$ for any t), we find that ψ^{X^t} converges to this stationary distribution $\tilde{\psi}^X$ monotonically in terms of the KL divergence $D_{\psi||\xi}(X^t)$. Similar proofs apply to continuous time Markov chains and diffusion, since we only need to replace φ by ξ and repeat the derivation leading to Eq. (S50) and Eq. (S67).

In the two regimes discussed above in Sec. S8A, the population state X corresponds to some fixed genotype G or a vector of allele frequencies P. Therefore $D_{\psi||\xi}(X^t) = D_{KL}(\psi^{G^t}||\tilde{\psi}^G)$ or $D_{\psi||\xi}(X^t) = D_{KL}(\psi^{P^t}||\tilde{\psi}^P)$ are non-increasing functions of time. Together with Eq. (S77,S79), this implies that F^G or F^P are non-decreasing functions of time.

Iwasa (6) and Sella and Hirsh (10) proved the same result by different methods. In our framework it emerges as a special case of the information accumulation bound with zero control. The key part of our proof, stating that $D_{\psi||\xi}(X^t)$ is non-increasing, is also more general (regarding the state space, the form of the stationary distribution, and detailed balance – although free fitness is not defined so generally). A similarly general proof for continuous time Markov chains, as well as several related results for replicator dynamics and reaction networks, is reviewed in reference (14).

S8D. Free fitness as a trade-off between fitness and information. We can rewrite the expressions for free fitness using the genotype and population-level information respectively. We first write down the neutral stationary distributions. On the genotype level, we assume that it is uniform over 4^l possible sequences of length l,

$$\tilde{\varphi}^G(g) = \frac{1}{4^l}. \quad \text{[S83]}$$

On the population level,

$$\tilde{\varphi}^P(p) = \frac{1}{Z^{\varphi,P}} \prod_i (p_i q_i)^{2N\mu - 1}, \quad \text{[S84]}$$

where $Z^{\varphi,P}$ is the normalization constant, to be distinguished from Z^P in Eq. (S75) which includes selection.

Using $\tilde{\varphi}^G(g)$ and $\tilde{\varphi}^P(p)$, we can rewrite free fitness, Eq. (S77,S79), as

$$F^G = \underbrace{\langle \ln w_g \rangle_{\psi^G}}_{\text{Selection}} - \frac{1}{2N} \underbrace{\left\langle \ln \frac{\psi^G(g)}{\tilde{\varphi}^G(g)} \right\rangle_{\psi^G}}_{\substack{\text{Genotype-level}\\ \text{information}}} - \underbrace{\frac{1}{2N} \ln(4^l)}_{\substack{\text{Independent}\\ \text{of } \psi^G}} = \langle \ln w_g \rangle_{\psi^G} - \frac{1}{2N} D(G) + \text{const.} \quad \text{[S85]}$$

on the genotype level and

$$F^P = \underbrace{\langle \ln \bar{w}(p) \rangle_{\psi^P}}_{\text{Selection}} - \frac{1}{2N} \underbrace{\left\langle \ln \frac{\psi^P(p)}{\tilde{\varphi}^P(p)} \right\rangle_{\psi^P}}_{\substack{\text{Population-level}\\ \text{information}}} + \underbrace{\frac{\ln Z^{\varphi,P}}{2N}}_{\substack{\text{Independent}\\ \text{of } \psi^P}} = \langle \ln \bar{w}(p) \rangle_{\psi^P} - \frac{1}{2N} D(P) + \text{const.} \quad \text{[S86]}$$

on the population level. In both cases we have emphasized that terms independent of ψ^G or ψ^P are constant in time and therefore not important for the dynamics of free fitness. Up to the constant, this formula for free fitness has also been used in the paper on fitness flux (15). The fitness flux theorem (ref. (15) and Sec. S10) then provides perhaps the most elegant proof that free fitness is a non decreasing function of time, as it relates changes in $D(P)$ to changes in expected fitness.

Free fitness tends to increase over time until it is maximized at the Boltzmann-like equilibrium distribution $\tilde{\psi}^G$ or $\tilde{\psi}^P$. In other words, evolution maximizes the expected log fitness while constraining the amount of genetic information, with $1/(2N)$ serving as a Lagrange multiplier that controls the trade-off.

S9. Properties of measures of cost of selection

Here we prove general inequalities between the genetic load $L(x)$, relative fitness variance $V(x)$, and the information theoretic cost $C(x)$. We also derive the form of $C(x)$ for the special cases of weak selection and truncation selection. The three measures are defined as

$$L(x) = 1 - \frac{1}{\hat{w}_{\max}(x)} \qquad \text{[S87]}$$

$$V(x) = \sum_g x_g \left(\hat{w}_g(x) - 1\right)^2, \qquad \text{[S88]}$$

$$C(x) = \sum_g x_g \hat{w}_g(x) \log_2 \hat{w}_g(x), \qquad \text{[S89]}$$

where x_g is the frequency of genotype g in the population and $\hat{w}_{\max}(x) = \max_{g;\, x_g>0} \hat{w}_g(x)$ is the relative fitness of the fittest individual that is present in the population ($x_g > 0$).

We note that some previous work has defined $\hat{w}_{\max}(x)$ to be the maximum fitness possible, i.e. the fitness of an ideal genotype with no deleterious mutations regardless of whether such an individual exists. Load computed with such a definition is higher, and this has led to claims of severe restrictions on the rate of adaptive substitutions (16) and the functional fraction of the human genome (17). However, load under this definition has been criticized as irrelevant, since the ideal genotype has a vanishing probability of existing in the population, and if only the fitness values likely to be present in the population are considered, load-based restrictions are more permissive (18, 19). Our definitions of $L(x)$, $V(x)$ and $C(x)$ all focus on the existing variation of fitness in the population x. $L(x)$ is also related to the concept of lead, which was defined as the difference between the maximum and the mean log fitness in a traveling wave (20).

Truncation selection and limitations by reproductive capacity. Under truncation selection, a fraction α of individuals in the population has constant relative fitness, equal to the maximum $\hat{w}_g(x) = \hat{w}_{\max}(x)$ and the remaining fraction $1-\alpha$ has relative fitness zero $\hat{w}_g(x) = 0$. By definition, the mean relative fitness must be $\sum_g x_g \hat{w}_g(x) = 1$, which requires $\hat{w}_{\max}(x) = 1/\alpha$. The three measures of cost of selection then are

$$L^{\text{trunc}}(x) = 1 - \alpha, \qquad \text{[S90]}$$

$$V^{\text{trunc}}(x) = \alpha \left(\frac{1}{\alpha} - 1\right)^2 + (1-\alpha)(0-1)^2 = \frac{1}{\alpha} - 1, \qquad \text{[S91]}$$

$$C^{\text{trunc}}(x) = -\log_2 \alpha. \qquad \text{[S92]}$$

At a constant population size, the expected number of offspring of an individual is equal to their relative fitness $\hat{w}_g(x)$ (or $2\hat{w}_g(x)$ under sexual reproduction, with two parents per offspring). In a species with a reproductive capacity R, we have $\hat{w}_{\max}(x) \leq R$ and the load is limited as $L \leq 1 - 1/R$. $V(x)$ and $C(x)$ at given R are maximized under truncation selection, when only the most extreme relative fitness values available are occupied ($N\alpha$ individuals have relative fitness $\hat{w}_g(x) = 1/\alpha = \hat{w}_{\max}(x) = R$ and $N - N\alpha$ individuals have fitness 0). This implies upper bounds $V(x) \leq R - 1$ and $C(x) \leq \log_2 R$.

General inequality between $L(x)$ and $V(x)$. From Eq. (S87), the genetic load $L(x)$ determines the maximum relative fitness in the population, $\hat{w}_{\max}(x) = 1/(1 - L(x))$. Given that relative fitness of all individuals in the population must lie between 0 and $\hat{w}_{\max}(x)$, its variance $V(x)$ is maximized when only these extreme values are occupied, i.e. under truncation selection. In that case we have $V^{\text{trunc}}(x) = 1/\alpha - 1$ with $\alpha = 1/\hat{w}_{\max}(x)$. This implies a general bound,

$$V(x) \leq V^{\text{trunc}}(x) = \frac{L(x)}{1 - L(x)}, \qquad \text{[S93]}$$

with equality under truncation selection. The same inequality was derived in ref. (21) by other means.

General inequality between $L(x)$ and $C(x)$. Since logarithm is an increasing function and $x_g \hat{w}_g(x) \leq 0$, we can upper bound each term of the form $x_g \hat{w}_g(x) \log_2 \hat{w}_g(x)$ in Eq. (S89) by $x_g \hat{w}_g(x) \log_2 \hat{w}_{\max}(x)$. Summing over g, we obtain

$$C(x) \leq \sum_g x_g \hat{w}_g(x) \log_2 \hat{w}_{\max}(x) = \log_2 \hat{w}_{\max}(x) = \log_2 \frac{1}{1 - L(x)}. \qquad \text{[S94]}$$

Equality is again achieved under truncation selection.

General inequality between $V(x)$ and $C(x)$. We use the inequality $\log_2 u \leq \frac{u-1}{\ln 2}$ in Eq. (S89) to obtain

$$C(x) \leq \sum_g x_g \hat{w}_g(x) \frac{\hat{w}_g(x) - 1}{\ln 2} = \frac{1}{\ln 2} \left(\sum_g x_g \hat{w}_g(x)^2 - 1 \right) = \frac{V(x)}{\ln 2}. \qquad \text{[S95]}$$

Equality is approached under truncation selection when $\alpha \to 1$.

$C(x)$ under weak selection. Here we assume that for all genotypes g present in the population ($x_g > 0$), the relative fitness $\hat{w}_g(x)$ is close to 1. We can then use the Taylor expansion

$$\hat{w}_g(x)\log_2 \hat{w}_g(x) = \frac{1}{\ln 2}\left(\hat{w}_g(x) - 1 + \frac{1}{2}\left(\hat{w}_g(x) - 1\right)^2 + O\left(\left(\hat{w}_g(x) - 1\right)^3\right)\right). \tag{S96}$$

Combining this with Main Text Eq. (S89), we find

$$C(x) = \frac{1}{\ln 2}\sum_g x_g\left(\hat{w}_g(x) - 1 + \frac{1}{2}\left(\hat{w}_g(x) - 1\right)^2 + O\left(\left(\hat{w}_g(x) - 1\right)^3\right)\right) \tag{S97}$$

$$= \frac{V(x)}{2\ln 2} + \frac{1}{\ln 2}\sum_g x_g\, O\left(\left(\hat{w}_g(x) - 1\right)^3\right), \tag{S98}$$

or in short, $C(x) \approx V(x)/(2\ln 2)$ under weak selection. This is particularly relevant for the diffusion limit, when the population size is sent to infinity, and the selection strength is rescaled inversely to the population size.

S10. Fitness flux theorem

In this section we compare the newly introduced bound on information accumulation rate (the *cost of selection bound*) and a similar bound implied by the fitness flux theorem (15) (the *fitness flux bound*). The fitness flux theorem was originally derived under the diffusion approximation. For better comparison, we also derive an analogous result for discrete-time Markov chains. We then discuss the distinct interpretation of the two bounds, and illustrate them (using both the discrete and diffusion expressions) in Fig. S2.

S10A. Discrete-time Markov chains. The fitness flux theorem, like its counterparts in statistical physics (e.g. (22)), is based on the comparison of forward and reverse path probabilities. For simplicity, we will not derive the fitness flux theorem in its general form, but rather the form that allows a direct comparison with the cost of selection bound.

We focus on short paths consisting of only one step, (X^t, X^{t+1}). The probability of the forward path (x^t, x^{t+1}) is $\psi^{X^t}(x^t)P(x^{t+1}|x^t)$. We consider a probability distribution over reverse paths, $\psi^{X^{t+1}}(x^{t+1})P(x^t|x^{t+1})$, which is normalized to 1 – we can write this as

$$1 = \sum_{x^t\, x^{t+1}} \psi^{X^t}(x^t)\, P(x^{t+1}|x^t) \exp\ln\frac{\psi^{X^{t+1}}(x^{t+1})\, P(x^t|x^{t+1})}{\psi^{X^t}(x^t)\, P(x^{t+1}|x^t)} \tag{S99}$$

By Jensen's inequality,

$$0 \geq \sum_{x^t\, x^{t+1}} \psi^{X^t}(x^t)\, P(x^{t+1}|x^t)\ln\frac{\psi^{X^{t+1}}(x^{t+1})\, P(x^t|x^{t+1})}{\psi^{X^t}(x^t)\, P(x^{t+1}|x^t)}, \tag{S100}$$

Next, inside the logarithm, we divide and multiply by the neutral probabilities,

$$0 \geq \sum_{x^t\, x^{t+1}} \psi^{X^t}(x^t)\, P(x^{t+1}|x^t)\ln\frac{\psi^{X^{t+1}}(x^{t+1})\, P(x^t|x^{t+1})\,\frac{\varphi^{X^{t+1}}(x^{t+1})\,Q(x^t|x^{t+1})}{\varphi^{X^{t+1}}(x^{t+1})\,Q(x^t|x^{t+1})}}{\psi^{X^t}(x^t)\, P(x^{t+1}|x^t)\,\frac{\varphi^{X^t}(x^t)\,Q(x^{t+1}|x^t)}{\varphi^{X^t}(x^t)\,Q(x^{t+1}|x^t)}} \tag{S101}$$

$$= \sum_{x^t\, x^{t+1}} \psi^{X^t}(x^t)\, P(x^{t+1}|x^t)\ln\frac{\frac{\psi^{X^{t+1}}(x^{t+1})\,P(x^t|x^{t+1})}{\varphi^{X^{t+1}}(x^{t+1})\,Q(x^t|x^{t+1})}}{\frac{\psi^{X^t}(x^t)\,P(x^{t+1}|x^t)}{\varphi^{X^t}(x^t)\,Q(x^{t+1}|x^t)}}, \tag{S102}$$

where we assumed that the neutral process is at a stationary distribution with detailed balance, i.e. $\varphi^{X^t}(x^t)\,Q(x^{t+1}|x^t) = \varphi^{X^{t+1}}(x^{t+1})\,Q(x^t|x^{t+1})$. Finally, we rearrange terms and divide by $\ln 2$ to get an expression in bits,

$$\text{Fitness flux bound:}\quad \Delta D(X^t) \leq \sum_{x^t}\psi^{X^t}(x^t)\sum_{x^{t+1}} P(x^{t+1}|x^t)\log_2\frac{P(x^{t+1}|x^t)\,Q(x^t|x^{t+1})}{Q(x^{t+1}|x^t)\,P(x^t|x^{t+1})} = 2N\langle\phi\rangle_t, \tag{S103}$$

$$\text{Cost of selection bound:}\quad \Delta D(X^t) \leq \sum_{x^t}\psi^{X^t}(x^t)\sum_{x^{t+1}} P(x^{t+1}|x^t)\log_2\frac{P(x^{t+1}|x^t)}{Q(x^{t+1}|x^t)} \leq kN\langle C\rangle_t, \tag{S104}$$

where $\langle\phi\rangle_t$ is the discrete analog of the fitness flux, averaged over the possible transitions (x^t, x^{t+1}),

$$\phi(x^t, x^{t+1}) = \frac{1}{2N}\log_2\frac{P(x^{t+1}|x^t)\,Q(x^t|x^{t+1})}{Q(x^{t+1}|x^t)\,P(x^t|x^{t+1})}. \tag{S105}$$

The interpretation of this expression and the relationship to fitness accumulation is most clear in the diffusion approximation, see below. To help compare the fitness flux bound with the cost of selection bound, we take the expectation over the final state

x^{t+1} and compute the expected fitness flux from any initial state x^t, $\phi(x^t) = \sum_{x^{t+1}} P(x^{t+1}|x^t)\phi(x^t, x^{t+1})$. An example plot of $\phi(x^t)$ is in Fig. S2A. For comparison, we also included in Eq. (S104) the cost of selection bound.

While the cost of control is a non-negative conditional KL divergence, the fitness flux bound contains an additional term related to the reverse transition probabilities, and can be negative (this is more easily interpretable as the mutation term in the diffusion approximation). The fitness flux bound relies on the additional assumption that the neutral process is at a stationary distribution with detailed balance. This can be satisfied in the single locus, two allele system when using the Moran model, but it is violated by the Wright-Fisher model which we use throughout most of the paper.

S10B. Diffusion approximation. Mustonen and Lässig (15) derive the fitness flux theorem using a similar method but under the diffusion approximation, where the fitness flux is related to the rate accumulation of fitness. We include here an informal account of how that relates to the formula in Eq. (S104).

In continuous time, we can generalize the definition of fitness flux in Eq. (S105) to an arbitrary time interval Δt and the transition $(x^t, x^{t+\Delta t})$.

$$\phi(x^t, x^{t+\Delta t}) = \frac{1}{\Delta t}\frac{1}{2N}\log_2 \frac{P(x^{t+\Delta t}|x^t)\, Q(x^t|x^{t+\Delta t})}{Q(x^{t+\Delta t}|x^t)\, P(x^t|x^{t+\Delta t})}, \qquad \text{[S106]}$$

where we also divided by Δt to get the fitness flux per generation. Under the diffusion approximation, if Δt is small, the transition probabilities P, Q will be approximately normal with parameters given by $a(x^t)$, $a^s(x^t)$ and $b(x^t)$ (see Sec. S7; we drop the dependence on x^t for brevity),

$$P(x^{t+\Delta t}|x^t) \approx \mathcal{N}(x^{t+\Delta t};\, x^t + a + a^s,\, b), \qquad \text{[S107]}$$

$$Q(x^{t+\Delta t}|x^t) \approx \mathcal{N}(x^{t+\Delta t};\, x^t + a,\, b). \qquad \text{[S108]}$$

Then in Eq. (S106) we recover the definition of fitness flux from (15),

$$\phi(x^t, x^{t+\Delta t}) \approx \frac{1}{N\Delta t \ln 2}\,(x^{t+\Delta t} - x^t)_g\, b^{-1}_{gg'}\, a^s_{g'} \qquad \text{[S109]}$$

where we sum over repeated indices as in Sec. S7. Note that the factor $1/\ln 2$ appears because we use base 2 logarithms throughout. If the process takes place in a fitness landscape/seascape F, the vector $b^{-1}_{gg'}\, a^s_{g'}/N = \partial_g F$ is its gradient, and $\phi(x^t, x^{t+\Delta t})$ is the rate at which the system climbs it up and therefore accumulates fitness (Fig. 1 and Eq. S8-S10 in (15)). This interpretation of fitness flux is exact in the diffusion approximation as $\Delta t \to 0$, but only approximate for the discrete formulas in Eq. (S105) and Eq. (S103).

We can take the expectation over $x^{t+\Delta t}$ to obtain the expected fitness flux per generation from any starting position x^t,

$$\phi(x^t) \approx \frac{1}{N\ln 2}(a + a^s)_g b^{-1}_{gg'}\, a^s_{g'}, \qquad \text{[S110]}$$

which is also plotted in Fig. S2A. Finally, we can take the expectation over x^t to obtain the fitness flux bound in the diffusion approximation. It compares with the cost of selection bound as follows,

$$\text{Fitness flux bound:} \quad \frac{d}{dt}D(X^t) \le \frac{2}{\ln 2}\int \psi\,\left(a_g + a^s_g\right) b^{-1}_{gg'}\, a^s_{g'}\, dx = 2N\langle\phi\rangle_t, \qquad \text{[S111]}$$

$$\text{cost of selection bound:} \quad \frac{d}{dt}D(X^t) \le \frac{1}{2\ln 2}\int \psi\, a^s_g\, b^{-1}_{gg'}\, a^s_{g'}\, dx \qquad \le N\langle C\rangle_t. \qquad \text{[S112]}$$

Note that in Eq. (S111) we added the factor 2 which was missing in (15), as pointed out in (23).

Again, the fitness flux bound requires the neutral process to be at a stationary distribution with detailed balance, which means zero neutral flux $u_g = 0$ (see Eq. (S57)). (There is a more general flux theorem, which does not require detailed balance (15), but it does not provide a bound on $\Delta D(X^t)$.) The single locus, two allele system satisfies the detailed balance, since diffusion only takes place along a single dimension. However, detailed balance is rare in systems with multiple loci with recombination and general forms of mutation.

S10C. Comparisons of the discrete and the diffusion formulas. The two bounds, computed using both the Markov chain and the diffusion formulas, are compared in Fig. S2BC for the single locus, two allele system. Note that the bounds are obtained by averaging the functions $2N_e\phi(x_A)$ and $N_eC(x_A)$, such as those plotted in Fig. S2A, with respect to the distribution $\psi^X(x_A)$.

In Fig. S2B, we use the Wright-Fisher model to compute the distribution over allele frequencies and the information increments. The population size is $N_{\mathrm{WF}} = N_e = 100$, mutation strength $N\mu = 0.01$ and selection strength varies across columns. The cost of selection bound is in black, and the diffusion formula (full line, based on Eq. (S112)) is in an agreement with the discrete formula (dashed line, based on Eq. (S104)). The discrete version of the fitness flux bound is violated, as the Wright-Fisher model does not satisfy detailed balance (grey dashed, based on Eq. (S103)). This is because cycles such as $0 \to 1 \to 2 \to 0$ copies of the A allele take place more often than the reverse cycle, since two alleles can get lost by drift in a single generation with a high probability, but are unlikely to arise by mutation. However, detailed balance holds in the diffusion approximation. If fitness flux is computed according to the diffusion formula, the bound holds under weak selection

when the diffusion approximation is close to the Wright-Fisher model, but fails when selection is stronger (full grey line, based on Eq. (S111)).

In Fig. S2C, we use the Moran model of the same system to compute the distributions and information increment over time. Note that in order to have the same magnitude of genetic drift as the Wright-Fisher model, the Moran model needs twice as large a census population size, $N_{\text{Moran}} = 2N_e$. Time is measured in generations ($N_{\text{Moran}} = 2N_e$ replacements each), and the increments in information are also computed per generation.

The discrete and diffusion formulas are again in agreement for the cost of selection bound. Note that we computed it as $N_e\langle C\rangle_t$ rather than $N_{\text{Moran}}\langle C\rangle_t$, to account for the additional stochasticity due to random deaths, even though $N_{\text{Moran}} = 2N_e$ parents are sampled with selection in each generation. The fitness flux bound now holds in its discrete version – the Moran model only allows allele frequency changes by $\pm 1/N_{\text{Moran}}$ and therefore satisfies detailed balance. If fitness flux is computed using the diffusion formula in Eq. (S111), it upper bounds the Moran model accumulation of information when selection is weak, but again fails when selection is strong and diffusion departs from the discrete model.

In conclusion, care is needed when applying the fitness flux bound to discrete models. In Main Text Fig. 4, we plot the information accumulation and the cost of selection bound based on the Wright-Fisher model, and the fitness flux bound based on the Moran model.

S10D. Interpretation of the bounds under diffusion. The cost of control is non-negative and determined solely by the magnitude of the selection term a_g^s, with the inverse drift covariance $b_{gg'}^{-1}$ acting as the metric. As shown in Sec. S7 and Fig. S2A, this is proportional to the variance in fitness,

$$\frac{d}{dt}D(X^t) \leq N\langle C\rangle_t \approx \frac{N}{2\ln 2}\langle V\rangle_t. \quad \text{[S113]}$$

In Fig. S2BC, this bound starts off large and reduces slightly as selection removes variation from the population. It remains positive at the stationary state, where it represents the cost of maintenance.

In contrast, the fitness flux bound in Eq. (S111) contains the sum $a_g^s + a_g$, corresponding to selection and mutation contributions to the fitness flux. As a result, the fitness flux bound can be written as a sum of a selection term and a mutation term,

$$\frac{d}{dt}D(X^t) \leq 2N\langle\phi\rangle_t = \underbrace{\frac{2N}{\ln 2}\langle V\rangle_t}_{\text{Selection term}} + \underbrace{\frac{2}{\ln 2}\int \psi\, a_g\, b_{gg'}^{-1}\, a_{g'}^s\, dx}_{\text{Mutation term}}. \quad \text{[S114]}$$

The selection term is proportional to the fitness variance – like the cost of selection bound, but with a 4 times higher numerical coefficient. When the selection term in Eq. (S114) dominates (a regime also discussed in (15)), the two bounds are proportional to each other, but the cost of selection bound is tighter. In general, the mutation term in Eq. (S114) causes the two bounds to behave in qualitatively different ways. Notably, the mutation term can be substantial and comparable to the selection term even when the parameters N, s, μ suggest that selection is strong and mutation is weak (i.e. under any or all of the conditions $Ns \gg 1$, $N\mu \ll 1$ and $s \gg \mu$). We illustrate and explain this in the following paragraphs.

Fig. S2A shows that the selection term in Eq. (S114) dominates especially at intermediate allele frequencies (around $x_A = 0.5$), where the fitness variance is high and mutation in opposing directions cancels out. Near $x_A = 0$, fitness variance is low and mutation towards the fitter allele A leads to a positive expected fitness flux. Near $x_A = 1$, mutation towards the deleterious allele a dominates and leads to a negative expected fitness flux (Fig. S2A).

This enables a more detailed understanding of the fitness flux bound in the scenario in Main Text Fig. 4 and Fig. S2C. The system is initialized at the neutral stationary distribution, which is symmetric. Therefore the mutation term in fitness flux vanishes, because the positive and negative contributions (at $x_A < 0.5$ and $x_A > 0.5$ respectively) cancel out. The bound is therefore proportional to the average fitness variance. Over time, as selection shifts the distribution towards higher x_A, mutation contributes more and more negatively, until the mutation and selection terms exactly cancel at stationarity. This happens regardless of N, μ and s. At stationarity, populations fluctuate around frequencies where mutation and selection are balanced, typically close to $x_A = 0$ or $x_A = 1$.

The only regime when the selection term dominates fitness flux for an extended period of time is when not only mutation is weak overall, but also the population is initialized at an intermediate frequency. This is shown in Fig. S3, which uses the same parameters as Fig. S2C, but the population is initialized at the frequency $x_A = 0.5$.

Note that only the process with selection can be initialized at $x_A = 0.5$. The neutral process must always be at the neutral stationary distribution to satisfy the assumptions of the fitness flux theorem. As a result, the information $D(X^t)$ is very high initially and decreases until drift spreads out the distribution ψ^{X^t} towards $x_A = 0$ and 1. Later, $D(X^t)$ slightly increases over a much longer (mutation-limited) time scale, see Fig. S3A. The early phase is useful for illustrating the behavior of the fitness flux and the cost of selection, but neither bound is very informative there, as information is being lost.

In Fig. S3B we plot the increments in information, the fitness flux bound and the cost of selection bound per generation. Both bounds are proportional to the relative fitness variance in the early phase at intermediate frequencies, albeit with different proportionality constants (full and dotted purple lines in Fig. S3B). In the later phase, when the population is mostly fixed for one of the alleles, the mutation term in Eq. (S114) becomes important and negative, and the fitness flux bound departs from the fitness variance approximation.

S11. Frequency dependent selection that maximizes fixation probability

In Main Text Fig. 3C,D, we compare the efficiency of selection (accumulated information per unit cost of selection) under constant selection, and under a specific form of frequency dependent selection. This frequency dependence is optimal in the sense that it maximizes the fixation probability of A at a given cumulative cost of selection. Below we describe the optimization procedure.

The calculation is done using a single locus, two allele Wright-Fisher model as described in Sec. S4, but instead of a single selection coefficient, we have a vector $\boldsymbol{s} = \left(s\left(0\right), s\left(\frac{1}{N}\right), s\left(\frac{2}{N}\right), \ldots, s\left(1\right)\right)^\intercal$ of selection coefficients $s(x_A)$ for each possible allele frequency x_A. Given $\boldsymbol{s}$, we can compute

- The (right stochastic) transition matrix $P(\boldsymbol{s})$ according to Eq. (S10), using the respective selection coefficient for each starting frequency (rows of $P(\boldsymbol{s})$).
- The vector $\boldsymbol{\psi}^{\text{fix}}(\boldsymbol{s})$ of fixation probabilities for each possible starting frequency. It is given by the last column of the matrix power $P(\boldsymbol{s})^t$ at infinite time $t \to \infty$. Numerically, we keep doubling t, until the total probability that neither allele is fixed is less than 10^{-6} for all starting frequencies.
- The vector $\boldsymbol{c}(\boldsymbol{s})$ of cost of selection at each frequency, $\boldsymbol{c}(\boldsymbol{s}) = \left(C\left(0\right), C\left(\frac{1}{N}\right), C\left(\frac{2}{N}\right), \ldots, C\left(1\right)\right)^\intercal$. Note that $C(0) = C(1) = 0$ since there is no fitness variation when one of the alleles is fixed.
- The vector $\boldsymbol{\gamma}(\boldsymbol{s})$ of expected total cost of selection until either allele is fixed, for each starting frequency. The first and last elements are again equal to zero, since one of the alleles is fixed and no more cost is incurred. The remaining elements can be computed using the recurrence relation

$$\boldsymbol{\gamma}(\boldsymbol{s}) = \boldsymbol{c}(\boldsymbol{s}) + P(\boldsymbol{s})\boldsymbol{\gamma}(\boldsymbol{s}), \tag{S115}$$

  where $\boldsymbol{c}(\boldsymbol{s})$ is the immediate cost and $P(\boldsymbol{s})\boldsymbol{\gamma}(\boldsymbol{s})$ is the expected future cost. Eq. (S115) can be solved for $\boldsymbol{\gamma}(\boldsymbol{s})$ as a system of linear equations.
- The value vector $\boldsymbol{v}(\boldsymbol{s}) = \boldsymbol{\psi}^{\text{fix}}(\boldsymbol{s}) - \lambda\boldsymbol{\gamma}(\boldsymbol{s})$, where λ is the Lagrange multiplier which quantifies the constraint on the total cost.

We look for $\boldsymbol{s}$ which maximizes value $\boldsymbol{v}(\boldsymbol{s})$. This is an instance of a Markov decision process (24) similar to the pursuit/first passage problem (25) with a small modification to include an unwanted absorbing state (loss of the A allele). The frequency-dependent selection $\boldsymbol{s}$ corresponds to the decision policy. The optimal policy does not depend on time or the initial state, and maximizes all elements of $\boldsymbol{v}(\boldsymbol{s})$ simultaneously (25).

We optimize $\boldsymbol{s}$ iteratively. It is initialized at all zeros, and we alternate between a value update and a policy (selection) update,

$$\text{Value update:} \qquad \boldsymbol{v} := \boldsymbol{v}(\boldsymbol{s}) = \boldsymbol{\psi}^{\text{fix}}(\boldsymbol{s}) - \lambda\boldsymbol{\gamma}(\boldsymbol{s}) \tag{S116}$$

$$\text{Policy update:} \qquad \boldsymbol{s} := \operatorname{argmax}_{\boldsymbol{s}}\left(P(\boldsymbol{s})\boldsymbol{v} - \lambda\boldsymbol{c}(\boldsymbol{s})\right) \tag{S117}$$

The value update uses the current estimate of $\boldsymbol{s}$. The policy update uses the current estimate of $\boldsymbol{v}$ to compute the selection coefficient at each frequency, which maximizes the expected value in the next step, minus the immediate cost. The maximization is independent for each element of $\boldsymbol{s}$ and is done by binary searching for zero gradient ($\log_{10} s$ in range from 10^{-5} to 10^2, binary search depth 10).

To produce Fig. 3C,D, we vary the cost constraint λ and compute $\boldsymbol{s}$ by 60 value-policy update iterations. Examples of the frequency dependent $\boldsymbol{s}$ are shown in Fig. S4A. Notably, selection is strongest at low frequencies of x_A when A is at the greatest risk of being lost, but weak at higher frequencies to reduce costs. Fig. S4B,C show the fixation probability $\boldsymbol{\psi}^{\text{fix}}(\boldsymbol{s})$ and the expected total cost $\boldsymbol{\gamma}(\boldsymbol{s})$ for each starting frequency, from which the Main Text Fig. 3C,D uses only the values for the initial frequency $1/N$.

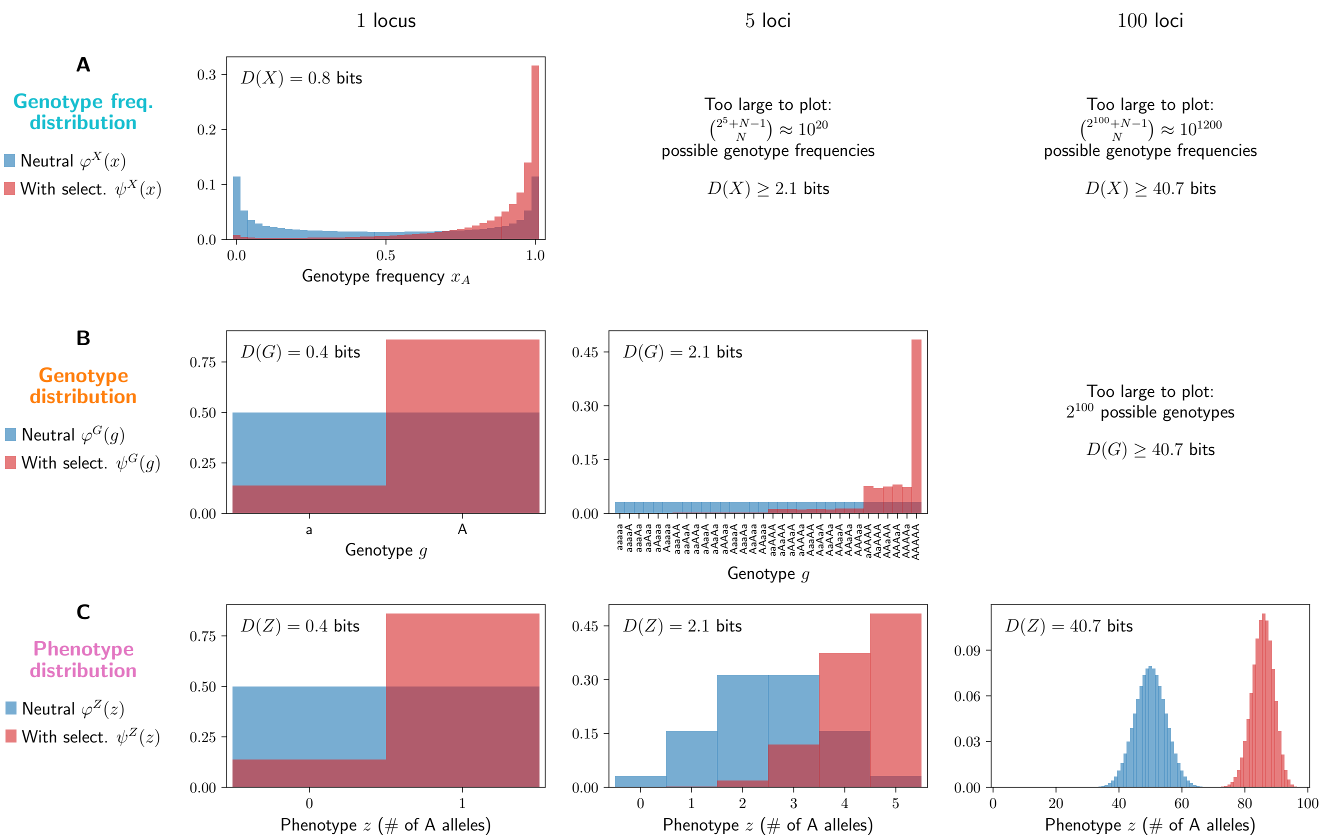

Fig. S1. An example of the distributions over genotype frequencies, genotypes and phenotypes, with and without selection and for a varying number of loci, along with the corresponding measures of information. Based on a Wright-Fisher model with parameters $N = 40$, $\mu = 0.005$, $s = 0.05$ (as in Main Text Fig. 1). Fitness is multiplicative across loci, $w = (1+s)^z$ where z is the number of beneficial A alleles carried – i.e. there is directional selection for an additive, fully heritable phenotype z (as in Main Text Fig. 6). All distributions are at the stationary state, computed using a transition matrix-based model (1 locus) or a long run (10^5 generations after 200 generations of burn-in) of an individual-based model (> 1 locus).

(A) Distributions over genotype frequencies ($\psi^X(x)$ with selection and $\varphi^X(x)$ without, red and blue) and the population-level information, $D(X)$. The distributions cannot be plotted and $D(X)$ cannot be directly estimated for 5 and 100 loci, due to the large number of possible genotype frequencies x, but we can still lower bound $D(X)$ by $D(G)$ or $D(Z)$.

(B) Distributions over genotypes ($\psi^G(g)$ with selection and $\varphi^G(g)$ without, red and blue) and the genotype-level information, $D(G)$. This information is less than $D(X)$, because selection not only gives preference to the fitter alleles, but also reduces the genetic variation within populations. The number of possible genotypes becomes too large for 100 loci, but we can still lower bound $D(G)$ by $D(Z)$.

(C) Distributions over the phenotype ($\psi^Z(z)$ with selection and $\varphi^Z(z)$ without, red and blue) and the phenotype-level information, $D(Z)$. The phenotype is simply the number of A alleles across all loci in an individual – an additive trait of varying polygenicity. In this example, selection favors the A allele at each locus, making fitness a function of z. In such cases, $D(G) \approx D(Z)$, since grouping genotypes into bins of equal z reduces the state space but preserves selective differences. In general, the trait might be unrelated to fitness or form only a component of it, leading to $D(G) > D(Z)$. Note that $D(Z)$ is approximately proportional to the number of loci, with each locus encoding about 0.4 bits. This is because loci evolve approximately independently, as there is zero epistasis, free recombination and little Hill-Robertson interference (see also Main Text Sec. 4.2-4.3).

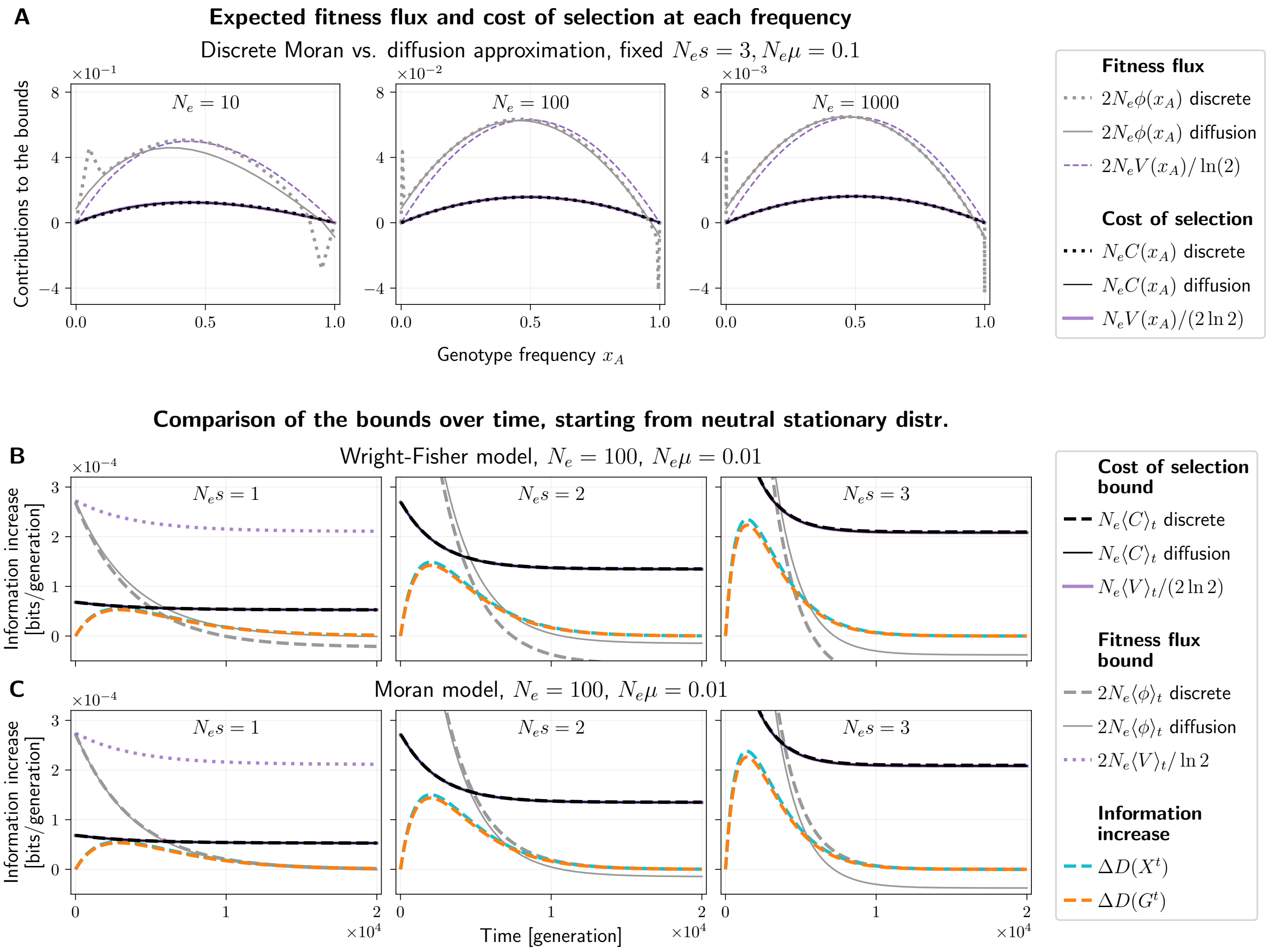

Fig. S2. Comparisons between the fitness flux, the information-theoretic cost of selection and fitness variance, using the single locus, two allele systems under the Wright-Fisher model, the equivalent discrete-time Moran model and the diffusion approximation.

(A) We compute the expected fitness flux per generation ($\phi(x_A)$, gray dotted) and the cost of selection ($C(x_A)$, black dotted) for each frequency x_A under the discrete Moran-model for three different effective population sizes N_e (left to right; census population size $N_{\mathrm{Moran}} = 2N_e$ to account for the additional stochasticity in the Moran model compared to the Wright-Fisher model). We fixed $N_e s = 3$ and $N_e\mu = 0.1$ to obtain models with a similar behavior but different time scales and granularity. Both $\phi(x_A)$ and $C(x_A)$ are multiplied by N_e and the same numerical factor as in the bounds on information accumulation rate (the bounds are obtained by averaging these values with respect to $\psi^X(x_A)$). The discrete formulas are compared with their diffusion approximation (full gray and black lines). As expected, the diffusion approximation is closer to the discrete model at higher N_e. The fitness flux $\phi(x_A)$ converges to the diffusion approximation non-uniformly across x_A, due to the spikes next to the domain boundaries. These spikes do not disappear but get "squeezed out" at high N_e. We also plot multiples of the relative fitness variance $V(x_A)$ (purple dashed and full lines), which approximate the fitness flux and the cost of selection. The cost $C(x_A)$ can be approximated very closely by $V(x_A)/(2\ln 2)$ as long as $s \ll 1$ (here, the largest value is $s = 0.3$ for $N_e = 10$). Fitness flux is the sum of a selection term proportional to $V(x_A)$ which is largest at intermediate x_A, and a mutation term which dominates near $x_A = 0$ or 1 and causes the discrepancy between $\phi(x_A)$ and $2V(x_A)/\ln 2$. Even when mutation rate is small compared to selection, $\mu \ll s$, mutation is important as the system approaches the stationary distribution concentrated near $x_A = 0$ and 1.

(B) The Wright-Fisher model uses the same parameters as Main Text Fig. 4, namely $N = N_e = 100$; $N_e\mu = 0.01$ and $N_e s$, varied across columns. We plot the increase of information per generation (population level $\Delta D(X^t)$, blue dashed; genotype level $\Delta D(G^t)$, orange dashed), the upper bound in terms of cost of selection $N_e\langle C\rangle_t$, computed using the discrete formula (Eq. (S104), black dashed) and the diffusion formula (Eq. (S112), black full), and the upper bound in terms of the fitness flux computed using the discrete formula (Eq. (S103), gray dashed) and the diffusion formula (Eq. (S111), gray full). We also show the fitness variance approximations of the two bounds (Eq. (S113), purple solid and the fitness variance term in Eq. (S114), purple dotted, outside the plot range for $N_e s = 2$ and 3). The discrete fitness flux bound is violated, since the Wright-Fisher model does not satisfy detailed balance under neutrality. The continuous fitness flux bound holds under weak selection, but fails when selection gets stronger, as differences grow between the discrete system and the diffusion approximation.

(C) The Moran model has the same effective parameters but double the census population size, $N_{\mathrm{Moran}} = 2N_e$. The curve descriptions are the same as for the Wright-Fisher model, but note that each generation consists of $2N_e$ replacements, and the information increase as well as the upper bounds are rescaled accordingly. Also note that we plot the cost of selection bound $N_e\langle C\rangle_t$ using the effective population size N_e rather than the census population size $N_{\mathrm{Moran}} = 2N_e$, which would lead to a twice as large (and less tight) bound. The Moran morel satisfies detailed balance under neutrality, and the discrete fitness flux bound holds under arbitrary selection. The continuous fitness flux bound again fails under strong selection.

Note that the diffusion formula for the fitness flux bound would correctly upper bound the accumulation of information in a system modeled fully using the diffusion approximation. These figures show that it does not always upper bound the accumulation of information in discrete models, especially not when selection is strong. The Main Text Fig. 4 uses the information accumulation curves and cost of selection bounds based on the Wright-Fisher model, and the fitness flux bound based on the Moran model.

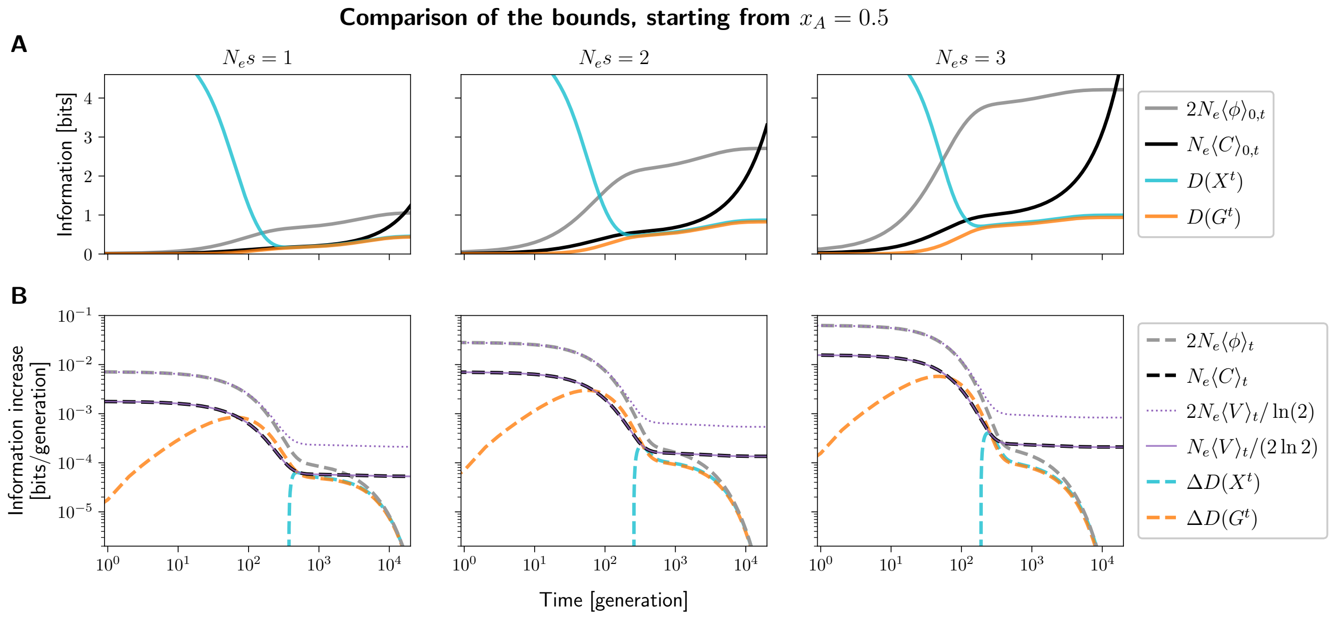

Fig. S3. Demonstration of the fitness flux and the cost of selection bounds in the single locus, two allele system initialized at an intermediate frequency $x_A = 0.5$. Note that only the process with selection is initialized at $x_A = 0.5$; the neutral process must always be at the neutral stationary distribution to satisfy the assumptions behind the fitness flux theorem. This leads to a high initial value of $D(X^t)$ at $t = 0$. Calculated using the discrete Moran model with the same parameters as in Fig. S2C.

(A) The cumulative information at the population level ($D(X^t)$, blue) and genotype level ($D(G^t)$, orange), as well the cumulative fitness flux ($2N_e\langle\phi\rangle_{0,t}$, gray) and cumulative cost of selection ($N_e\langle C\rangle_{0,t}$, black). $D(X^t)$ starts out high due to the initial distribution being different under selection than under neutrality ($D(X^0) \approx 12.3$ bits, outside the plot range, in all three cases). At first, drift causes the distribution under selection to spread out towards extreme frequencies, $D(X^t)$ decreases on the time scale of $\sim N_e = 100$ generations. Meanwhile, $D(G^t)$ accumulates as the mean frequency x_A increases from 0.5 due to selection. The two measures of information eventually reach similar values, since mutation is low and the population is mostly fixed for one of the alleles. The cumulative fitness flux and cost of selection upper bound the cumulative information increase $D(X^t) - D(X^0)$, but are not very informative in this case, because despite selection acting, $D(X^t)$ started at a value that is higher than can be maintained and is lost rather than accumulated.

(B) The increase of information per generation on the population and genotype levels ($\Delta D(X^t)$, blue dashed; $\Delta D(G^t)$, orange dashed), and the upper bounds in terms of fitness flux ($2N_e\langle\phi\rangle_t$, gray dashed) and the cost of selection ($N_e\langle C\rangle_t$, black dashed). Note the log scales on both axes. Initially, $\Delta D(X^t)$ is negative and falls outside of the plot as drift spreads out the distribution over allele frequencies. Meanwhile, $\Delta D(G^t)$ is positive as the mean frequency x_A increases due to selection – this is associated with a positive fitness flux and cost of selection. After about $2N_e = 200$ generations, one of the alleles is likely to be fixed by drift, but the mean frequency continues to slowly increase at a mutation-limited rate (time scale $1/\mu = 10^4$ generations), which also causes modestly positive $\Delta D(X^t)$ and $\Delta D(G^t)$. In this phase, the cost of selection remains nearly constant, while fitness flux slowly decays, providing a fairly tight bound on $\Delta D(X^t)$. The cost of selection bound can be very well approximated with the relative fitness variance ($N_e\langle V\rangle_t/(2\ln 2)$, purple solid line). The fitness flux is proportional to the fitness variance in the first phase, when x_A is near 0.5 ($2N_e\langle V\rangle_t/\ln 2$, purple dotted line), but departs from it later as x_A tends to take values near 0 or 1 where mutation is important (see Fig. S2A). We note that similar behavior can also observed for different values of $N_e\mu$, $N_e s$.

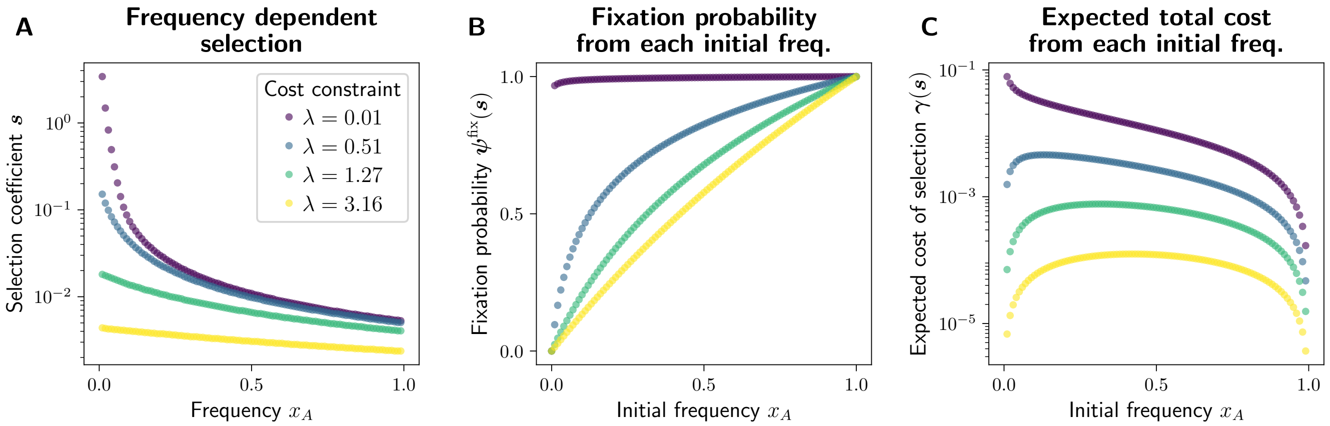

Fig. S4. Frequency dependent selection that optimizes fixation probability of an allele at a constrained total cost.
(A) The frequency dependent selection coefficient s, computed as described in Sec. S11, under various cost constraints λ. When this constraint is greater, selection is weaker overall.
(B) Fixation probability $\psi^{\rm fix}(s)$ of the allele A for each starting frequency x_A. The fixation probability is close to the frequency itself under weak selection (large λ), and higher when selection is overall stronger.
(C) The expected total cost of selection, $\gamma(s)$ associated with trajectories starting from each initial frequency x_A. It is low for high frequencies, where the allele A is expected to be fixed soon, and for low frequencies under weak selection, where it is expected to be lost soon.

References

1. TM Cover, JA Thomas, *Elements of Information Theory.* (John Wiley & Sons), Second edition, (2006).
2. RP Worden, A speed limit for evolution. *J. Theor. Biol.* **176**, 137–152 (1995).
3. E Todorov, Linearly-solvable Markov decision problems in *Advances in Neural Information Processing Systems.* Vol. 19, pp. 1369–1376 (2006).
4. EA Theodorou, Nonlinear stochastic control and information theoretic dualities: Connections, interdependencies and thermodynamic interpretations. *Entropy* **17**, 3352–3375 (2015).
5. G Kesidis, J Walrand, Relative entropy between Markov transition rate matrices. *IEEE Transactions on Inf. Theory* **39**, 1056–1057 (1993).
6. Y Iwasa, Free fitness that always increases in evolution. *J. Theor. Biol.* **135**, 265–281 (1988).
7. H Hasegawa, Thermodynamic properties of non-equilibrium states subject to Fokker-Planck equations. *Prog. Theor. Phys.* **57**, 1523–1537 (1977).
8. CS Withers, S Nadarajah, The spectral decomposition and inverse of multinomial and negative multinomial covariances. *Braz. J. Probab. Stat.* **28**, 376–380 (2014).
9. J Berg, S Willmann, M Lässig, Adaptive evolution of transcription factor binding sites. *BMC Evol. Biol.* **4**, 42 (2004).
10. G Sella, AE Hirsh, The application of statistical physics to evolutionary biology. *Proc. Natl. Acad. Sci.* **102**, 9541–9546 (2005).
11. S Wright, The distribution of gene frequencies in populations. *Proc. Natl. Acad. Sci.* **23**, 307–320 (1937).
12. HP de Vladar, NH Barton, The contribution of statistical physics to evolutionary biology. *Trends Ecol. & Evol.* **26**, 424–432 (2011).
13. R Rao, S Leibler, Evolutionary dynamics, evolutionary forces, and robustness: A nonequilibrium statistical mechanics perspective. *Proc. Natl. Acad. Sci.* **119**, e2112083119 (2022).
14. JC Baez, BS Pollard, Relative entropy in biological systems. *Entropy* **18**, 46 (2016).
15. V Mustonen, M Lässig, Fitness flux and ubiquity of adaptive evolution. *Proc. Natl. Acad. Sci.* **107**, 4248–4253 (2010).
16. M Kimura, Evolutionary rate at the molecular level. *Nature* **217**, 624–626 (1968).
17. D Graur, An upper limit on the functional fraction of the human genome. *Genome Biol. Evol.* **9**, 1880–1885 (2017).
18. WJ Ewens, Remarks on the substitutional load. *Theor. Popul. Biol.* **1**, 129–139 (1970).
19. B Galeota-Sprung, P Sniegowski, W Ewens, Mutational load and the functional fraction of the human genome. *Genome Biol. Evol.* **12**, 273–281 (2020).
20. MM Desai, DS Fisher, Beneficial mutation–selection balance and the effect of linkage on positive selection. *Genetics* **176**, 1759–1798 (2007).
21. EE Shnol, EA Ermakova, AS Kondrashov, On the relationship between the load and the variance of relative fitness. *Biol. Direct* **6**, 20 (2011).
22. GE Crooks, Path-ensemble averages in systems driven far from equilibrium. *Phys. Rev. E* **61**, 2361–2366 (2000).
23. NH Barton, How does epistasis influence the response to selection? *Heredity* **118**, 96–109 (2017).
24. R Bellman, A Markovian decision process. *J. Math. Mech.* **6**, 679–684 (1957).
25. JH Eaton, LA Zadeh, Optimal pursuit strategies in discrete-state probabilistic systems. *J. Basic Eng.* **84**, 23–29 (1962).
